# Two bromodomain proteins functionally interact to recapitulate an essential BRDT-like function in *Drosophila* spermatocytes

**DOI:** 10.1098/rsob.140145

**Published:** 2015-02-04

**Authors:** Shuhei Kimura, Benjamin Loppin

**Affiliations:** Centre de Génétique et de Physiologie Moléculaire et Cellulaire, CNRS UMR5534, Université Claude Bernard Lyon 1, 69622 Villeurbanne cedex, France

**Keywords:** bromodomain and extra terminal family, *Drosophila*, tBRD-1, tBRD-2, spermatocyte

## Abstract

In mammals, the testis-specific bromodomain and extra terminal (BET) protein BRDT is essential for spermatogenesis. In *Drosophila*, it was recently reported that the tBRD-1 protein is similarly required for male fertility. Interestingly, however, tBRD-1 has two conserved bromodomains in its N-terminus but it lacks an extra terminal (ET) domain characteristic of BET proteins. Here, using proteomics approaches to search for tBRD-1 interactors, we identified tBRD-2 as a novel testis-specific bromodomain protein. In contrast to tBRD-1, tBRD-2 contains a single bromodomain, but which is associated with an ET domain in its C-terminus. Strikingly, we show that *tbrd-2* knock-out males are sterile and display aberrant meiosis in a way highly similar to *tbrd-1* mutants. Furthermore, these two factors co-localize and are interdependent in spermatocytes. We propose that *Drosophila* tBRD-1 and tBRD-2 associate into a functional BET complex in spermatocytes, which recapitulates the activity of the single mammalian BRDT-like protein.

## 2. Introduction

Bromodomains are highly conserved domains known to recognize acetylated lysine residues of histones and other proteins [1]. Members of the BET family of nuclear proteins typically have two bromodomains in their N-terminus associated with one extra terminal (ET) domain in their C-terminus [2]. The ET domain is considered as a protein–protein interacting domain [3] although its function remains unknown. Members of the BET family of proteins are generally well conserved in many species from yeast to human [2]. In mammals, there are four BET proteins, BRD2, BRD3, BRD4 and BRDT, which are well studied for their important roles as transcriptional co-regulators and cell-cycle regulators [4]. Recently, BET proteins were also identified as promising drug targets for cancer treatment [5], and BRDT was notably proposed as a potential target for male contraception [6]. Mammalian BRDT is a testis-specific factor essential for spermatogenesis [7]. Functional studies in mice have revealed that BRDT plays widespread roles at different stages of spermatogenesis, including transcriptional regulation, meiosis, chromocentre organization, mRNA splicing and 3′UTR-truncation [8–11]. On the other hand, it has been shown that BRDT has the capacity to remodel chromatin in an acetylation-dependent manner [12], and crystal structure analysis revealed that bromodomain 1 of BRDT specifically recognizes multi-acetylated histone H4 tails [13]. These data raised the attractive hypothesis that BRDT recognizes and removes hyper-acetylated histones before protamine incorporation and genome compaction during spermiogenesis [11].

The founding member of the BET family of proteins is encoded by the *female sterile* (*1*) *homeotic* (*fs*(*1*)*h*) gene identified in *Drosophila* [14,15]. The Fs(1)h protein, which is ubiquitously expressed, has been shown to activate homeotic genes during embryogenesis [16,17]. In *Drosophila*, two testis-specific genes, *testis-specifically expressed bromodomain-containing protein-1* (*tbrd-1*) [18] and *mitoshell* (*mtsh*) [19] were reported to express bromodomain proteins essential for spermatogenesis. In contrast to Mtsh that has one bromodomain-related region in which the ligand-binding residues are not conserved, tBRD-1 contains two bromodomains related to BET protein bromodomains; bromodomain 1 of tBRD-1 is the closest to bromodomain 1 of Fs(1)h using BLASTP analysis (data not shown). These features open the possibility that tBRD-1 could represent the functional equivalent of mammalian BRDT in flies. However, the tBRD-1 protein, which is shorter than Fs(1)h, lacks an ET domain in its C-terminus and hence cannot be definitively classified into the BET family of proteins.

Here, in an attempt to clarify the role of tBRD-1 during spermatogenesis, we searched for tBRD-1 protein partners using biochemical approaches and identified a novel testis-specific protein, CG7229/tBRD-2. We show that tBRD-2 is also a bromodomain protein, but which additionally contains an ET domain. tBRD-1 and tBRD-2 are both essential for male fertility, and their respective mutants display remarkably similar meiotic defects. Finally, we demonstrate that tBRD-1 and tBRD-2 co-localize in spermatocyte nuclei and are interdependent, suggesting that these two factors collectively behave as a single BET protein, in a way reminiscent of BRDT in mammalian male germ cells.

## 3. Results and discussion

### 3.1. Generation of a *tbrd-1* null mutant

**Figure 1.**
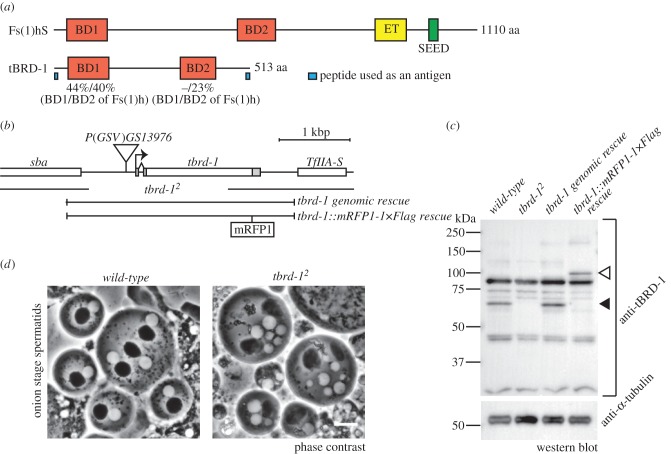
Generation of *tbrd-1* null mutant. (*a*) Representations of Fs(1)hS (short isoform of Fs(1)h) and tBRD-1 proteins. Bromodomain 1 (BD1) of tBRD-1 showed 44% identity with BD1 of Fs(1)h and 40% identity with bromodomain 2 (BD2) of Fs(1)h. BD2 of tBRD-1 showed 23% identity with BD2 of Fs(1)h, but no significant homology (−) with BD1 of Fs(1)h. Sequence identity was analysed using ClustalW. BD, bromodomain; ET, ET domain; SEED, seed motif. Blue boxes showed the peptides used as antigens for anti-tBRD-1 antibody generation. (*b*) A map of the *tbrd-1* gene locus. The deletion range in *tbrd*-*1^2^* and two rescue constructs (*tbrd-1 genomic rescue* and *tbrd-1::mRFP1-1×Flag rescue*) are shown. Triangle indicates the position of the *P*(*GSV6*)*GS13976* insertion mobilized to generate the *tbrd-1^2^* allele. (*c*) Western blotting analysis of tBRD-1 protein. Anti-α-tubulin antibody was used as control. *Wild-type* and *tbrd-1 genomic rescue* lanes showed endogenous tBRD-1 protein at about 65 kDa (black arrowhead), which is not detected in *tbrd-1^2^* extracts. In the *tbrd-1::mRFP1-1×Flag rescue* lane, a higher molecular weight band (white arrowhead) was observed, corresponding to mRFP1-1×Flag fused tBRD-1. (*d*) Phase contrast view of spermatids at onion stage. In *wild-type* (left panel), haploid nuclei (white circles) and mitochondrial derivatives, so-called Nebenkern (black circles), are all at the same size. By contrast, spermatids from *tbrd-1^2^* testes (right panel) contain nuclei of various sizes and irregularly shaped mitochondrial derivatives. Note that in these preparations, spermatids occasionally fuse during the course of live observation. Scale bar, 10 μm.

The tBRD-1 protein has two bromodomains, which are similar to the bromodomains of the Fs(1)h protein (figure 1*a*). Expression of *tbrd-1* mRNA in adult flies is essentially restricted to the testis according to the FlyAtlas database [20]. The previously reported *tbrd-1^1^* allele is a deletion of the C-terminal region but the truncated protein is apparently still expressed at low levels in the mutant [18]. Here, we recovered another *tbrd-1* allele (*tbrd-1^2^*) by imprecise excision [21] of a P-element inserted 137 bp upstream of the putative transcriptional start site (TSS) of the *tbrd-1* gene (figure 1*b*). The *tbrd-1^2^* allele removes a 1940 bp genomic region containing the TSS. Accordingly, *tbrd-1^2^* homozygous males showed complete sterility, although females were fertile (data not shown). Male sterility was rescued by two types of transgenic alleles; one contains the genomic region around the *tbrd-1* locus (*tbrd-1 genomic rescue*) and another one expresses tBRD-1 fused in frame with the monomeric red fluorescent protein1 (mRFP1) and 1× Flag tag (*tbrd-1::mRFP1-1×Flag rescue*). We also generated a rabbit polyclonal antibody against a mixture of two peptides from the very N- and C-terminal regions of tBRD-1, respectively. In western blotting (WB) experiments, this antibody recognized a band corresponding to tBRD-1 at about 65 kDa (predicted molecular weight is 59.2 kDa) in *wild-type* testicular extracts but not in *tbrd-1^2^* extracts. Furthermore, WB analysis of protein extracts from transgenic testes confirmed the specificity of this band (figure 1*c*). We conclude that *tbrd-1^2^* is a null or at least a strong loss of function allele. Phase contrast observation of mutant testes revealed that post-meiotic spermatid nuclei at the onion stage contained nuclei of various sizes associated with irregularly shaped mitochondria derivatives (figure 1*d*). This phenotype is indicative of abnormal chromosomal segregation and cytokinesis in meiosis [22]. After meiosis, although some spermatid cysts started to elongate, they appeared abnormal in shape and contained aberrant nuclei scattered throughout their length, in contrast to *wild-type* (data not shown). These phenotypes appeared very similar to what was previously reported for *tbrd-1^1^* by Leser *et al.* [18].

### 3.2. tBRD-1 interacts with a novel extra terminal domain-containing protein, CG7229/tBRD-2

**Figure 2.**
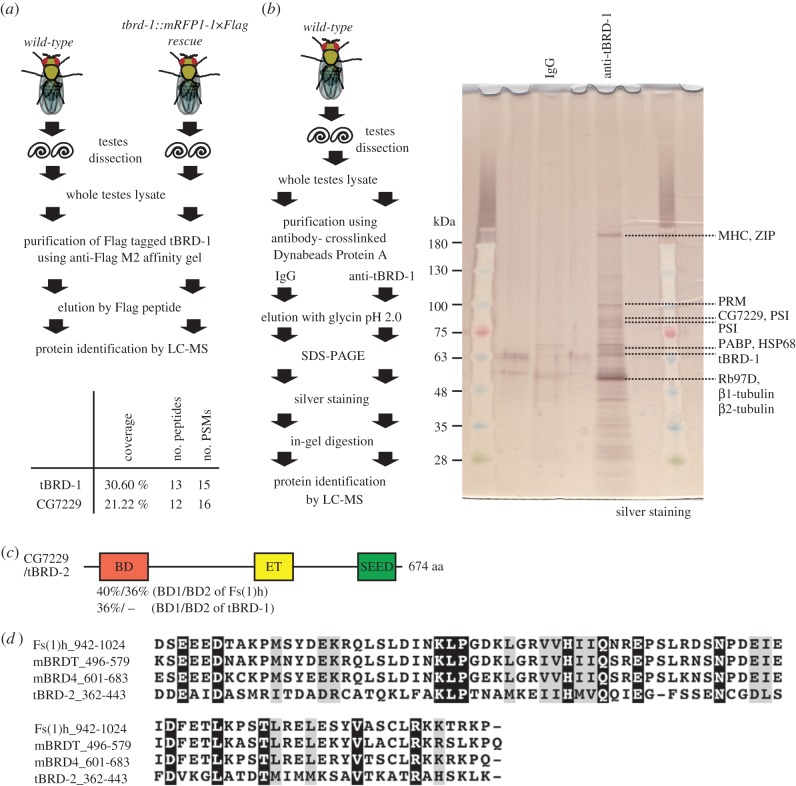
tBRD-1 interacts with a novel ET domain-containing protein, tBRD-2. (*a*) A scheme for the identification of the tBRD-1 interacting proteins using immunoprecipitation (IP) of exogenous tBRD-1. In the result table, coverage indicates the percentage coverage of identified polypeptides in full-length protein, no. peptides indicates the number of identified distinct peptides and peptide spectrum matches (no. PSMs) indicates the total number of identified peptides. (*b*) A schematic view of the identification of the tBRD-1 interacting proteins using IP of endogenous tBRD-1. SDS-gel was stained with the silver method. IgG lane was used as control. Proteins identified by LC-MS after in-gel digestion are indicated. MHC, myosin heavy chain; ZIP, zipper; PRM, paramyosin; PSI, P-element somatic inhibitor; PABP, poly(A) binding protein; HSP68, heat shock protein 68; Rb97D, ribonuclear protein at 97D. (*c*) Representation of CG7229/tBRD2 protein in *Drosophila*. BD of tBRD-2 showed 40% identity with BD1 of Fs(1)h and 36% identity with BD2 of Fs(1)h. It also showed 36% identity with BD1 of tBRD-1; however, it showed no significant homology (−) with BD2 of tBRD-1. Sequence identity was analysed using ClustalW. BD, bromodomain; ET, ET domain; SEED, seed motif. (*d*) Sequence alignment of ET domain using ClustalW. Black boxes and white lettering, identical amino acids; grey boxes and black lettering, same group of amino acids.

To gain insight about how tBRD-1 could exert its function in the male germline, we searched for tBRD-1 interacting proteins using immunoprecipitation followed by proteomic analyses. We conducted two different strategies for the immunoprecipitation procedures. First, we used *wild-type* testes and *tbrd-1::mRFP1-1×Flag rescue* testes (which express mRFP1 and 1×Flag-tagged tBRD-1 without endogenous tBRD-1; figure 2*a*). This recombinant protein is functional as it rescues the sterility of mutant males (data not shown). Crude testicular lysates were incubated into an anti-Flag M2 agarose affinity gel. Finally, total elution products were analysed by liquid chromatography–mass spectrometry (LC-MS). Interestingly, this first approach identified a remarkable abundance of peptides derived from the putative protein encoded by the *CG7229* gene, along with tBRD-1 peptides (electronic supplementary material, table S1). In a second approach, we directly used our anti-tBRD-1 antibody for immunoprecipitation using *wild-type* testicular lysates (figure 2*b*). Elution products were separated by electrophoresis and analysed by LC-MS. Again, this independent approach identified CG7229 as a potential tBRD-1 interacting protein (figure 2*b*; electronic supplementary material, table S2). As these independent immunoprecipitation strategies both identified CG7229 reproducibly as a putative binding partner of tBRD-1, we then focused on its characterization. Remarkably, CG7229 is also a bromodomain-containing protein, and like tBRD-1, is also specifically expressed in the adult testis, according to FlyAtlas [20]. However, CG7229 only has a single predicted bromodomain which shows 40% and 36% identity with the first and second bromodomains of Fs(1)h, respectively (figure 2*c*). Interestingly, the CG7229 protein has an ET domain characteristic of BET proteins (InterPro site annotated ‘NET domain (N-terminus of ET domain)’; N-terminal part of ET domain well-conserved in BET protein; figure 2*d*). Finally, this protein has a series of serine, aspartic acid and glutamic acid residues in the C-terminal, corresponding to the SEED motif as often seen in BET family of proteins (figure 2*c*) [7]. We also found that the *CG7229* coding region contained one polymorphism in the sequence (T1627C) compared with the reference sequence in Flybase. This substitution induces the replacement of serine 543 with a proline between the ET and SEED domains. While preparing this report, another group also identified *CG7229* as an interacting partner of tBRD-1, and named this gene *tbrd-2* (*testis-specifically expressed bromodomain-containing protein-2*) [23].

### 3.3. tBRD-2 is a spermatocyte-specific nuclear protein essential for male fertility

**Figure 3.**
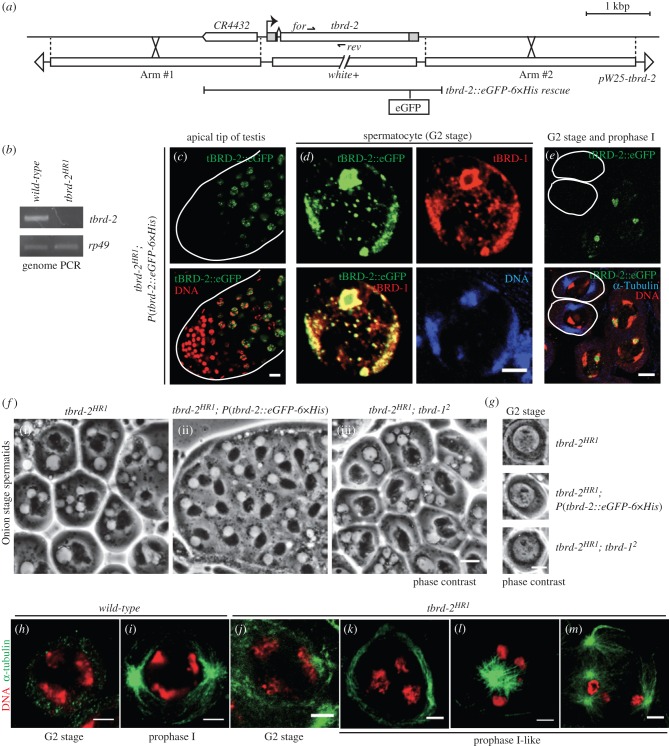
Meiotic defects in the *tbrd-2* mutant. (*a*) Schematic of the *tbrd-2* gene locus, the *pW25-tbrd-2* construct used for homologous recombination and the *tbrd-2::eGFP-6×His rescue* construct. (*b*) Genomic PCR analysis of the *tbrd-2* locus using the primers represented as ‘for’ and ‘rev’ in (*a*). *rp49* primers were used for control amplification. (*c*–*e*) Confocal images showing the localization of tBRD-2::eGFP in testes. (*c*) Apical tip of the testis. tBRD-2::eGFP gradually appeared during primary spermatocyte growth. The testis is outlined with a white line. Scale bar, 10 μm. (*d*) Mature primary spermatocytes. tBRD-1 and tBRD-2::eGFP signals are almost completely overlapping. Scale bar, 5 μm. (*e*) Mature primary spermatocytes and prophase I meiotic cells (outlined cells, characterized by the presence of two prominent asters and condensed chromatin). tBRD-2::eGFP is not detected in spermatocytes in prophase I. Scale bar, 10 μm. (*f*) Phase contrast view of post-meiotic spermatids at onion stage. *tbrd-2^HR1^*(i) and *tbrd-2^HR1^; tbrd-1^2^* double mutant (iii) present similar defects. Spermatids in the *tbrd-2::eGFP-6×His rescue* (ii) appear normal. Scale bar, 10 μm. (*g*) Phase contrast view of mature primary spermatocytes. Primary spermatocytes appear normal for all genotypes. Scale bar, 10 μm. (*h*–*m*) Confocal images of spermatocytes in meiosis I. Scale bar, 5 μm. (*h*,*i*) *Wild-type*: (*h*) G2 stage, (*i*) prophase I. (*j*–*m*) *tbrd-2^HR1^*: (*j*) G2 stage, (*k*–*m*) prophase I-like. (*k*) Although the chromatin appeared condensed, asters are not present. (*l*) Spermatocytes with asters in aberrant disposition. (*m*) Meiosis I with aberrant number of asters.

To examine the *in vivo* role of the *tbrd-2* gene, we generated *tbrd-2* mutant flies. As there was no available P-element insertion in or near the *tbrd-2* locus, we selected the homologous recombination method in order to obtain a precise deletion of the gene [24]. We indeed obtained a *tbrd-2* mutant allele, *tbrd-2^HR1^,* in which the *tbrd-2* gene was replaced by the *white* marker cassette from the recombination vector (figure 3*a*,*b*). *tbrd-2^HR1^* homozygous males were completely sterile (data not shown). This sterility was fully rescued by a transgene expressing tBRD-2 fused with enhanced green fluorescent protein (eGFP) and 6×Histidine tag (*tbrd-2*::*eGFP-6×His rescue*) under the control of its endogenous putative promoter. We also used this rescue line to study the localization of tBRD-2 protein during spermatogenesis. tBRD2::eGFP-6×His protein initially appeared in young primary spermatocytes, and its level increased during primary spermatocyte growth (figure 3*c*). Interestingly, tBRD2::eGFP-6×His was highly enriched in the nucleolus and additionally showed a weaker staining in the main chromatin masses of spermatocyte nuclei (figure 3*d*). This protein localization pattern was very similar to the distribution of tBRD-1 [18]. In fact, immunostaining of tBRD-1 in *tbrd-2::eGFP-6×His rescue* flies showed almost complete merge of the eGFP and tBRD-1 signals, supporting our previous proteomics data. tBRD2::eGFP-6×His then rapidly disappeared during the G2–M transition when two prominent asters appeared at the onset of chromosome condensation (figure 3*e*).

To investigate the cause of male sterility, *tbrd-2^HR1^* mutant testes were observed under phase contrast microscopy. *tbrd-2^HR1^* mutant testes showed the same phenotype as *tbrd-1^1^* [18] and *tbrd-1^2^* (figure 1*c*) mutant testes. Spermatids were produced but contained nuclei of various sizes and irregular mitochondria, and displayed aberrant cytokinesis at the onion stage (figure 3*f*(i)). By contrast, spermatogenesis exhibited normal appearance in rescued testes (figure 3*f*(ii)). Furthermore, the spermatid phenotype of *tbrd-2^HR1^;tbrd-1^2^* double mutants was not different form the phenotype of each *tbrd-1^2^* or *tbrd-2^HR1^* single mutant, suggesting that these two factors genetically function in the same pathway (figure 3*f*(iii)). In addition, the double mutant did not affect primary spermatocyte morphology at earlier stages (figure 3*g*). Taken together, these genetic and cytological data strongly suggest that tBRD-1 and t-BRD2 have highly related functions in spermatocyte nuclei.

### 3.4. Meiotic defects in the *tbrd-2* mutant

To dissect the details of meiosis in the *tbrd-2* mutant, we observed squashed testes under the confocal microscope. To distinguish the stages of meiosis, α-tubulin staining was used as a marker, and the chromosome morphology was also analysed [25]. In G2 phase, we were not able to find clear differences between *wild-type* and *tbrd-2^HR1^* mutant (figure 3*h*,*j*). However, at prophase I when chromatin started to condense into the typical three main chromosome masses, the two prominent asters of microtubules present in *wild-type* spermatocytes (figure 3*i*) were either not formed (figure 3*k*), aberrant in aspect (figure 3*l*) or present in excessive numbers in the *tbrd-2^HR1^* mutant (figure 3*m*). Subsequently, in metaphase I, anaphase I and telophase I in *tbrd-2^HR1^* mutant spermatocytes, sister chromatid segregation appeared abnormal (data not shown), as a likely consequence of aberrant position or number of asters in prophase I. Although *tbrd-2* mutant spermatocytes display severe meiotic defects in microtubule organization and chromosome behaviour, these phenotypes are likely to be indirect consequences of earlier defects as tBRD-2 is not detected during meiosis.

### 3.5. tBRD-1 and tBRD-2 proteins are interdependent

**Figure 4.**
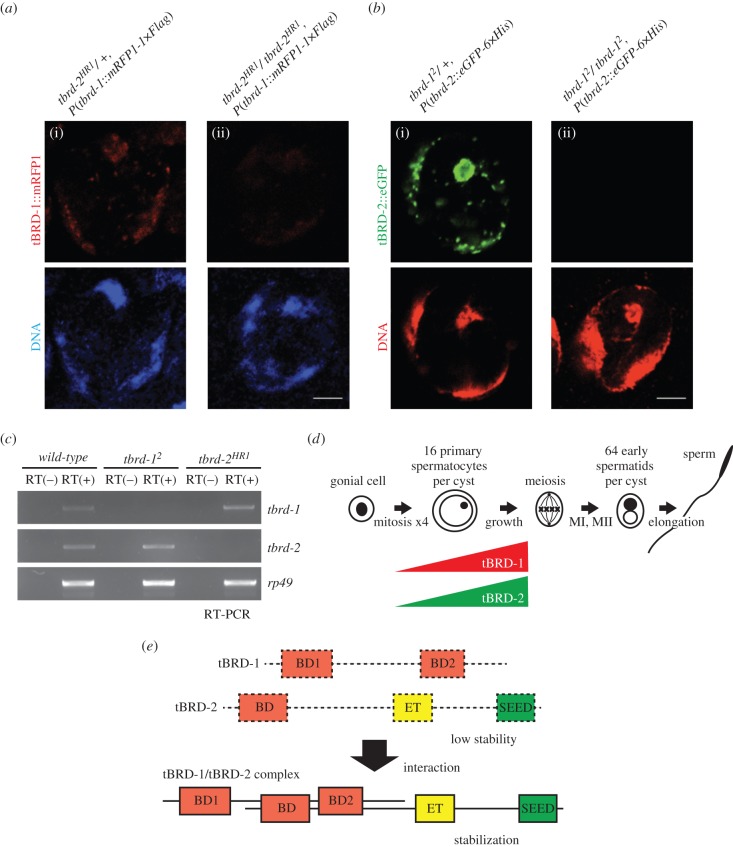
tBRD-1 and tBRD-2 interdependency in primary spermatocytes. (*a*,*b*) Confocal images. Scale bar, 5 μm. (*a*) tBRD-1::mRFP1 nuclear localization is dependent on tBRD-2. In *tbrd-2^HR1^* heterozygous (i), tBRD-1::mRFP1 is localized in nucleolus and chromatin. In *tbrd-2^HR1^* homozygous (ii), the tBRD-1::mRFP1 signal is strongly decreased. (*b*) tBRD-2::eGFP is dependent on tBRD-1. In *tbrd-1^2^* heterozygous (i), tBRD-2::eGFP is localized in nucleolus and chromatin. In *tbrd-1^2^* homozygous (ii), tBRD-2::eGFP signal is barely detected. (*c*) RT-PCR analysis. *tbrd-1* and tbrd-2 mRNA levels are not affected in *tbrd-2* and *tbrd-1* mutant testes, respectively. *rp49* transcripts were analysed as a control. RT, reverse transcriptase. (*d*) A scheme of the expression stage of tBRD-1 and tBRD-2 proteins in *Drosophila* spermatogenesis. Their expressions were limited in the primary spermatocyte. MI, meiosis I; MII, meiosis II. (*e*) A cooperation model for tBRD-1 and tBRD-2 proteins. Although tBRD-1 and tBRD-2 are unstable if they are alone, their interaction is required for their stability and the heterodimer functions as a single BRDT-like protein. BD, bromodomain; ET, ET domain; SEED, seed motif.

Since tBRD-1 and tBRD-2 appeared functionally related in our previous experiments, we wondered whether tBRD-1 and tBRD-2 proteins were dependent upon one another for their stability or distribution. First, we analysed the cellular localization of tBRD-1::mRFP1 in the *tbrd-2* mutant testes. In *tbrd-2* homozygous mutants, the tBRD-1::mRFP1 signal was strongly decreased compared to the *tbrd-2* heterozygous control (figure 4*a*). We also confirmed this result using the anti-tBRD-1 antibody (electronic supplementary material, figure S1). Conversely, we analysed the signal of tBRD-2::eGFP in *tbrd-1* homozygous mutant testes. Like the decrease of tBRD-1::mRFP1 signal, tBRD-2::eGFP was barely detected in *tbrd-1* homozygous mutant testes in contrast to the *tbrd-1* heterozygous control (figure 4*b*). Next, we tested whether they affected each other at the mRNA expression level. RT-PCR analysis indicated that *tbrd-1* and *tbrd-2* mRNA levels were not decreased in *tbrd-2* and *tbrd-1* mutants, respectively (figure 4*c*). In conclusion, these results suggest that tBRD-1 and tBRD-2 mutually depend on each other for their stability in growing primary spermatocytes (figure 4*d,e*).

These biochemical and genetic data support a functional partnership of tBRD-1 and tBRD-2 to ensure normal spermatogenesis in *Drosophila*. Gene fission is an evolutionary process in which an ancestral gene encoding a multi-domain protein splits into two complementary genes. It can occur through gene duplication followed by partial degeneration of protein domains in each duplicate [26]. For instance, such a scenario has been proposed for the evolution of the *cara mitad/Lost PHDs of trr* and *trithorax-related* (*trr*) genes, which are, respectively, orthologous to the N- and C-terminus of the mammalian transcriptional regulator MLL3 [27,28]. In the case of *tBRD-1* and *t-BRD2*, we hypothesize that these genes could have arisen after the evolutionary split of an ancestor *BRDT*-like gene. Indeed, tBRD-1 protein has two bromodomains characteristic of BET proteins but lacks an ET domain. On the other hand, tBRD-2 has a clear ET domain and SEED motif, but only a single bromodomain. Interestingly, with the exception of plant BET proteins that harbour a single bromodomain, members of this protein family always contain two bromodomains, from yeast to human [2]. We thus propose that the cooperation of these two factors could restore the functional equivalent of an ancestor BET protein, such as Fs(1)h, for instance. Although the tBRD-1/2 association cumulates three bromodomains, nothing is known about the actual functionality of each individual domain and this point will require additional analyses. We cannot exclude that one of these three potential bromodomains is actually in the process of degeneration. In addition, a recent report identified CG30417/tBRD-3 (testis-specifically expressed bromodomain-containing protein-3) as another testis-specific bromodomain and ET domain-containing protein [23]. Although tBRD-3 was shown to interact with tBRD-1 in a yeast two-hybrid assay, we could not identify any tBRD-3 peptides in our LC-MS analysis of tBRD-1::mRFP1-1×Flag interactors (electronic supplementary material, table S1). In any case, a functional analysis of tBRD-3 will be interesting to clarify the role of this factor during spermatogenesis.

In the case of mammalian BRDT, two types of functions were mainly reported [11,12]. One is a transcriptional regulator type of activity which relies on the recruitment of P-TEFb on target genes, in a way similar to BRD4. The other one is a chromatin remodeller type of activity based on the recognition of acetylated chromatin. These two types of activities seem to be exerted at different stages of spermatogenesis. In the case of *Drosophila* tBRD-1 and tBRD-2, their localization are restricted to primary spermatocytes. In these cells, tBRD-1 could function as a cofactor of testis-specific basic transcriptional factors (tTAFs), such as *spermatocyte arrest* (*sa*) [23,29]. On the other hand, our proteomic analyses of tBRD-1 interactors also identified PSI (RNA splicing protein) and Rb97D (mRNA binding protein; figure 2*b*). Interestingly, orthologues of these proteins (KH-type splicing regulatory protein (KHSRP) and heterogeneous nuclear ribonucleoprotein A1 (HNRNPA1), respectively) were previously identified as BRDT interacting proteins in mouse, among other RNA splicing factors [10]. Furthermore, a previous study aimed at identifying spliceosome associated factors in S2 cells listed up tBRD-1 itself [30]. It thus raises the intriguing possibility that tBRD-1 and tBRD-2 could be similarly involved in the splicing of meiosis-related mRNAs, which further supports the hypothesis that mammalian BRDT and *Drosophila* tBRD-1/2 could be functionally related.

Finally, our finding that two bromodomain factors essential for spermatogenesis probably reconstitute the functional equivalent of a BET protein supports the conserved need for a BRDT-like activity in animals.

## 4. Material and methods

### 4.1. Fly stocks

*w^1118^* and *y^1^w^67c^* stocks were used as *wild-type* strains. *P(GSV6)GS13976* stock was obtained from Drosophila Genetic Resource Center at Kyoto Institute of Technology. Other stocks were obtained from Bloomington *Drosophila* Stock Center. Flies were maintained on standard medium at 25°C.

### 4.2. Generation of mutant flies

#### 4.2.1. tbrd-1^2^

*P*(*GSV6*)*GS13976* is a *P*-element insertion 137 bp upstream of the putative *tbrd-1* TSS. After its mobilization with the P transposase expressing *Δ2–3* stock, we established 382 stocks for individual imprecise excision events. We identified three stocks with a genomic deletion covering the *tbrd-1* TSS site by genomic PCR and sequencing. All of three mutants showed the same phenotype, and we characterized in detail only one allele (*tbrd-1^2^*) in this report. The deletion range of *tbrd-1^2^* extends from 650 bp upstream to 1290 bp downstream of the putative TSS of *tbrd-1* gene.

#### 4.2.2. tbrd-2^HR1^

Approximately 3.0 kbp upstream (Arm#1) and downstream (Arm#2) of the *tbrd-2* gene locus were respectively amplified using *tbrd-2-arm#1-BsiWI-f*: CGCTTCGTACGCCAATGCATATTGCGCACAG and *tbrd-2-arm#1-AscI-r*: TAATTCGGCGCGCCCTAGTCAGGGATGCCAAGAC, *tbrd-2-arm#2-Acc65If*: TAATTCGGTACCACGATCACGTAGACCAGC and *tbrd-2-arm#2-NotI-r*: TAATTCGCGGCCGCCAGGATCGATAGCCTCGAG primers, and cloned into the *pW25* vector [31] with *BsiWI*, *AscI* and *Acc65I*, *NotI* restriction sites, respectively. *pW25-tbrd-2* transgenic flies were crossed with *y w/Dp*(*2;Y*)*G, P*(*hs-hid*)*Y; P{70FLP}23 P{70I-SceI}4A/TM3, P*(*hs-hid*)*14, Sb* stock [32] to perform *Ends-out* targeting. Among 303 individual F1 stocks, three lines showed the desired recombination event at the *tbrd-2* locus. The loss of *tbrd-2* in these alleles was confirmed by genomic PCR. All three *tbrd-2* alleles showed the same cytological defects in phase contrast microscopy, so we randomly selected one allele, *tbrd-2^HR1^*, for further characterization.

### 4.3. Transgenic flies

#### 4.3.1. Genomic tbrd-1

The genomic region from approximately 1.0 kbp upstream to approximately 0.5 kbp downstream of the *tbrd-1* gene locus was amplified with *tbrd-1-g1-KpnI-f*: GGGGTACCCCACAACAATTACATTTGCC and *tbrd-1-g2-NotI-r*: ATGCGCGGCCGCACCGAGAGGTGGCACCTTGG primers. PCR fragment was inserted into the *pW8* vector using *KpnI* and *NotI* restriction sites.

#### 4.3.2. Genomic tbrd-1::mRFP1-1×Flag

Approximately 1.0 kbp upstream and ORF of the *tbrd-1* gene was amplified using *tbrd-1-g1-KpnI-f* and *tbrd-1-g1-SalI-r*: ATGCGTCGACATCGCTATCATAAGTTTGGTCATCC. Approximately 0.5 kbp downstream of the *tbrd-1* gene including *tbrd-1* 3′UTR was amplified with *tbrd-1 g2-NotI-r* and *tbrd-1-g2-SpeI-f*: GGACTAGTAGTGTGTAGAATTCTACCACGG primers. These two fragments were inserted upstream and downstream of the mRFP1-1×Flag sequence in *pBS SK(+)* vector with *KpnI*/*SalI*, and *SpeI*/*NotI* sites, respectively, so that the mRFP1-1×Flag sequence was inserted into the C-terminus of the *tbrd-1* ORF immediately upstream of the stop codon. This construct was subcloned into the *pW8* vector using *KpnI* and *NotI* sites.

#### 4.3.3. Genomic tbrd-2::eGFP-6×His

Approximately, 1.0 kbp upstream and ORF of the *tbrd-2* gene was amplified with *tbrd-2-g1-SalI-f*: ATGTCGACATCGTAATATCTACGATGG and *tbrd-2-g1-XhoI-r*: ATGCTCGAGGGCCCTCAGCTGTCCTTCG primers. Approximately, 0.5 kbp downstream of the *tbrd-2* gene including *tbrd-2* 3′UTR was amplified with *tbrd-2-g2-SpeI-f*: ATGACTAGTTAATCATAAAGAAGTCTTATG and *tbrd-2-g2-NotI-r*: ATGCGGCCGCTTATGCAAATGAGAGTGC primers.

To make the eGFP-6×His sequence inserted into the C-terminus of the *tbrd-2* ORF immediately upstream of the stop codon, these two fragments were inserted upstream and downstream of the eGFP-6×His sequence using *SalI*, and *SpeI*/*NotI* sites. This construct was subcloned into the *pW8* vector with *XhoI* and *NotI* sites.

Additional plasmid construction details are available on request.

### 4.4. Fertility test

Five 0- to 1-day-old males were placed with five 0- to 1-day-old virgin females at 25°C. After 7 days, F0 flies were discarded, and the number of F1 larvae was counted.

### 4.5. Antibody generation

Two peptides from tBRD-1 (most N-terminus: MNELQSNSNQP+C, most C-terminus: C+EFSPGTEPLDDQTYDSD) were synthesized, and cysteine residues were conjugated with KLH (keyhole limpet haemocyanin). The mixture of these two peptides was used for the immunization of two rabbits. Crude sera were purified by immunoaffinity using the same peptides.

### 4.6. Immunoprecipitation

Preparation of crude protein lysate from testes. For each immunoprecipitation (IP), 200 pairs of testes were dissected in cold PBS and centrifugated at 3000 r.p.m. for 5 min at 4°C. After discarding the supernatant, 1×TNE buffer (20 mM Tris–HCl pH 7.5, 150 mM NaCl, 1 mM EDTA, 1% NP40) supplied with a protease inhibitor cocktail (Roche) was added, briefly sonicated and left for 10 min on ice. After centrifugation at 12 000 r.p.m. for 10 min at 4°C, supernatant was frozen in liquid nitrogen and kept at –80°C.

First IP: Flag M2 gel (Sigma #A2220) equilibrated with 1×TNE buffer was added to crude testicular lysates and rotated for 4 h at 4°C. After washing five times with 1×TNE buffer and substitution into 20 mM Tris–HCl pH 8.0 buffer, 1×Flag peptides (Sigma #F3290) in Tris–HCl pH 8.0 buffer were added and left for 30 min on ice. This elution step was done twice. Elution products were filtered using a concentration column (Millipore, Amicon ultra 0.5 ml, NMWL of 10 kDa) to remove the 1×Flag peptides. Samples were frozen in liquid nitrogen and stored at –80°C.

Second IP: Control rabbit IgG (Abcam #37415) and anti-tBRD-1 antibody were, respectively, added to Dynabeads Protein A (Invitrogen) and cross-linked using dimethyl pimelimidate-2HCl (DMP) (Thermo Scientific #21667). Antibody cross-linked Protein A Dynabeads were added to crude testicular lysates and rotated for 6 h at 4°C. After washing three times with 1×TNE buffer and once with BC100 (20 mM Hepes pH 7.6, 100 mM KCl, 0.2 mM EDTA, 10% glycerol, 0.05% Tween 20) buffer, 0.1 M glycin-HCl pH 2.0 was added and the mixture was left for 15 min at room temperature. 1 M Tris (with pH not adjusted) was rapidly added to the supernatant. Samples were frozen in liquid nitrogen and stored at –80°C.

### 4.7. Liquid chromatography–mass spectrometry

Total eluted proteins were precipitated with methanol–chloroform, trypsinized and peptides and were then analysed in LC-MS/MS using an LTQ Velos Orbitrap instrument (Thermo Fisher Scientific, Pittsburgh, PA, USA). In-gel digested peptides were also analysed by LC-MS/MS. To identify the proteins, spectra were analysed by Proteome Discoverer v. 1.2 (Thermo Scientific) against SEQUEST using a 5% false discovery rate (FDR) cutoff. Additional details are as previously described [33].

### 4.8. Western blotting

For each genotype, five pairs of testes were dissected in cold PBS and centrifuged at 3000 r.p.m. for 5 min at 4°C. After discarding the supernatant, 1× SDS sample buffer was added, and the samples were boiled for 10 min at 95°C. After brief centrifugation, samples were loaded on SDS-PAGE. WB was performed using standard procedures. Anti-α-tubulin (Sigma #T9026, mouse monoclonal, DM1A) was used at a 1/2000 dilution, anti-tBRD-1 was used at a 1/500 dilution. HRP-conjugated anti-mouse (Dako #P0161) and anti-rabbit (Dako #P0448) secondary antibodies were used at a 1/5000 dilution. Protein bands were detected using the ECL Plus WB Detection System (GE Healthcare).

### 4.9. Immunostaining

The testes squash and methanol–acetone fixation method was slightly adapted from Cenci *et al*. [25]. Testes were dissected in testis buffer (183 mM KCl, 47 mM NaCl, 10 mM Tris–HCl pH 6.8, 1 mM EDTA), and then opened from the tip using a fine needle. Dissected testes were placed into a drop of the testis buffer on poly-l-lysine-coated slides. Non-siliconized coverslips were put on the samples, the slides were inverted, squashed with moderate pressure and immediately placed in liquid nitrogen. After removing the coverslips, slides were immersed into methanol for 5 min at –20°C and subsequently in acetone for 2 min at –20°C. Next, samples were washed once with 1×PBST (1×PBS, 0.1% Triton X-100) buffer and incubated with primary antibodies in 1×PBST buffer overnight at 4°C. After three 1×PBST washes, secondary antibodies in 1×PBST were added overnight at 4°C. Finally, samples were washed three times, and propidium iodide (PI) or DRAQ5 containing DAKO mounting medium was added. For the PI staining, samples were incubated in 2 mg ml^−1^ RNase A in 1×PBST for 1 h at 37°C before mounting. Anti-α-tubulin (Sigma #T9026, mouse monoclonal, DM1A) was used at a 1/100 dilution, anti-tBRD-1 was used at a 1/100 dilution, DyLight 488 or 550-conjugated anti-mouse or rabbit antibody (Thermo Scientific) were used at a 1/200 dilution. PI (Sigma #P4170) and DRAQ5 (Cell Signalling Technology #4084L) were used to visualize DNA. Pictures were taken with an LSM 510 inverted confocal microscope (Zeiss). Figures were edited using the ImageJ software and Adobe Photoshop.

### 4.10. Phase contrast microscopy

Testes were dissected in testis buffer and opened using a fine needle. Testes were then observed on a slide with a coverslip placed without pressure. Live testes were observed using a Zeiss Axio inverted 2000 and Metaview v. 2 software. Figures were edited using the ImageJ software and Adobe Photoshop.

### 4.11. Genomic PCR and RT-PCR

Genomic DNA was extracted from a single fly using standard protocol. For cDNA preparation, 20 pairs of testes for each genotype were dissected, and total RNA was extracted using TRIzol reagent (Invitrogen) by standard protocol. From 70 ng total RNA, cDNA was produced using Super Script II reverse transcriptase (Invitrogen) by standard protocol. PCR for both genome and cDNA was performed using *Taq* DNA polymerase (5 PRIME) as follows: denaturation at 95°C for 1 min; annealing at 52°C for 1 min; elongation at 72°C for 1 min, 35 cycles. PCR primers were used as below:

*rp49-f*: AAGATCGTGAAGAAGCGCAC,
*rp49-r*: ACTCGTTCTCTTGAGAACGC,
*tbrd-1-f*: TGAAGCAATGGATCCACC,
*tbrd-1-r*: ACTCGCTGTTCTTGCACAAACAGG,
*tbrd-2-f*: TAGGACCAATGCTCCTAG,
*tbrd-2-r*: TGAGGATTCAATGCTAAC.

## Supplementary Material

Table S1

## Supplementary Material

Table S2

## Supplementary Material

Figure S1
