# Supplementary material for: Two bromodomain proteins functionally interact to recapitulate an essential BRDT-like function in Drosophila spermatocytes
Source: Open Biol. 2015 Feb 4;5(2):140145. doi: 10.1098/rsob.140145 (PMC4345279; doi:10.1098/rsob.140145)
Supplement: Table S1 [file rsob140145supp1.pdf]

Accession	Description	Score	Coverage	# Peptides	# PSMs	MW [kDa]	Peptide sequence	XCorr	Charge
gi7301065	CG13597 (tBRD-1)	67.65	30.60	13	15	59.21	ATESLDQASTSFSAPR	5.12	2
							KATESLDQASTSFSAPR	5.11	2
							MESIDLSTEVELKPK	4.75	2
							YYWQASEALEDFK	4.75	2
							LDSDNFESFDGFVSSVR	4.39	2
							QVTWAFNQADYWR	4.00	2
							mNELQSNSNQPPPR	3.84	2
							HPVDSVSLGVPDYHAVVK	3.59	2
							LLMEAFYMR	3.57	2
							TESLFVQEQR	3.26	2
							SVLNcLWR	2.68	2
							YRELIATAK	2.62	2
							YSQNPDYDHDREEK	2.61	2
							TESLFVQEQR	2.56	2
							HPVDSVSLGVPDYHAVVK	2.55	2
gi66771707	IP14655p (CG7229/tBRD-2)	63.55	21.22	12	16	74.59	NLPINSNPLQMQPIR	5.05	2
							SVNEFcLHVDGcFR	4.38	2
							SVNEFcLHVDGcFR	4.28	2
							LQSITNQTDAMTR	4.10	2
							SLVETFcDTLNK	3.73	2
							QIISNcFLFNR	3.53	2
							LTVNSLMQcVQMPGSGEPLTAK	3.49	2
							SVNEAIADFK	3.33	2
							cGPLINFR	3.18	2
							GLATDTMIMMK	3.18	2
							VQPEFIPHPGMAGR	3.11	2
							KLQSITNQTDAMTR	3.10	2
							QIISNcFLFNR	3.02	2
							GMPSGPEVPcNR	2.91	2
							LQSITNQTDAmTR	2.76	2
							cGPLINFR	2.76	2
gi113194667	smooth, isoform G	16.23	7.69	1	1	28.46	SSSGLIEFSNISQAVLAIMK	2.47	2
gi224967162	MIP08280p	15.73	18.78	3	3	25.06	LSYLVGLQMENFR	3.98	2
							IHNTGPTPVLAPSGLR	3.87	2
							VcPLcGVDEPEFHR	3.28	2
gi16198095	LD30988p	11.37	4.13	2	2	57.78	NVLMHGPPGTGK	3.22	2
							AQYQDQLSR	2.49	2
gi160714966	CG40813	10.73	14.55	2	2	24.41	LAELMNFVQVTNSQNHLEFLK	3.52	2
							GIIYISNIPK	2.87	2
gi21392218	RH43343p	10.13	13.16	2	3	17.62	YWQLTSSNLDSK	3.69	2
							YWQLTSSNLDSK	3.61	2
							VPNWFLNR	2.83	2
gi133771	40S ribosomal protein S14	7.85	15.89	2	2	16.25	IEDVTPIPSDSTR	2.99	2
							TPGPGAQSALR	2.73	2
gi51701866	60S ribosomal protein L10a-2	6.78	13.82	2	2	24.26	DTLYEGVNGLLEASAK	4.27	2
							VcILGDQQHcDEAK	2.50	2
gi25010003	GH11271p	4.93	1.96	1	3	98.23	RtYTVKNAVINMtLAQR	2.62	2
							RTyTVKNAVINMtLAQR	2.60	2
							RTYtVKNAVINMtLAQR	2.52	2
gi27819924	LD34308p	4.87	1.64	1	1	116.46	LPYELSSLDLTDFHtEK	2.81	2
gi15291475	GH23019p	4.50	3.59	1	1	59.18	gSPDsSPLLEKGRKPtIIK	2.54	2
gi158031869	CG34422, isoform B	4.45	2.88	1	1	67.19	yMDDRGTPLNKVPsILsR	2.61	2

gi21430004	GH24918p	3.71	2.84	1	1	52.10	LYEEQNKPEDAIR	3.71	2
gi19527923	AT15148p	3.54	4.98	1	1	33.67	ELGTQTAELPSQSQR	3.54	2
gi157816410	GH15225p	3.52	1.12	1	1	172.15	FASDNQEQDQQTAELQK	3.52	2
gi29335975	SD08737p	3.49	5.56	1	1	26.36	GLAVVDNSFQEVK	3.49	2
gi19527681	AT01812p	3.23	2.55	1	1	59.58	LDLYDSPDVDAWTR	3.23	2
gi23171693	GUK-holder, isoform A	3.11	1.62	1	1	190.95	VEPSTsAVSEYVSLNELPQHLRRHAVSSR	3.11	3
gi193883036	hypothetical protein CG32677	3.09	14.22	1	1	23.45	HtPLAAAISSTLQRSSPtSTITGNGIGNGSGK	3.09	3
gi113054	Acetylcholine receptor subunit alpha-like 2	3.05	3.13	1	2	65.46	VVWtPPAIFKsScEIDVR	3.05	2
							VVWtPPAIFKSscEIDVR	2.80	2
gi261259991	MIP14027p	2.96	5.83	1	1	27.53	LNALFDsKDLSGVK	2.96	2
gi41619982	TPA_inf: HDC16429	2.79	9.40	1	1	16.80	hLDSGcSPSILLtK	2.79	2
gi28317050	RE06501p	2.74	2.30	1	2	72.16	tPLQtYAVQIFHtLK	2.74	2
							tPLQTyAVQIFHtLK	2.51	2
gi16186138	SD06839p	2.73	4.42	1	1	43.88	tRVyVIGVGMtKFEKPGR	2.73	2
gi157816648	IP19953p	2.68	10.43	1	1	23.41	FLTILALAVASAsAYESVVHPK	2.68	2
gi15291685	LD23630p	2.62	2.80	1	1	62.39	LAPSQGTQSMAPPSVK	2.62	2
gi21064575	RE59324p	2.57	9.16	1	1	15.04	VTPDVVFAFGFR	2.57	2
gi10726715	cap-n-collar, isoform B	2.52	3.85	1	1	85.11	AVQQANYGGGVGVGVGVGVGVGSGTGsAFQR	2.52	2
gi157400268	CG2264, isoform F	2.52	3.27	1	1	59.08	rLASHQIGyNPDTSASK	2.52	2
gi74869876	Tubulin glycylase 3A	2.52	2.02	1	1	112.18	tPPPSKScPLGAPTNYVARR	2.52	2
gi25012817	RE74040p	2.48	1.98	1	1	113.03	tRVLGmLLNDAEVSRTQGSR	2.48	2
gi302176494	RpL22-like short isoform	2.48	14.63	1	1	12.75	VTPVETATPSAAPPLSSK	2.48	2
gi23172397	CG18472	2.47	2.77	1	1	53.79	sNLVSAAEALGNK	2.47	2
