# Supplementary material for: Two bromodomain proteins functionally interact to recapitulate an essential BRDT-like function in Drosophila spermatocytes
Source: Open Biol. 2015 Feb 4;5(2):140145. doi: 10.1098/rsob.140145 (PMC4345279; doi:10.1098/rsob.140145)
Supplement: Table S2 [file rsob140145supp2.pdf]

MHC, ZIP											
Accession	Description	Score	Coverage	# Peptides	# PSMs	MW [kDa]	Peptide sequence	# PSMs	XCorr	Charge
22946659	myosin heavy chain, isoform C (MHC)	246.32	31.96	57	77	224.28	GREEQAEQDGEEEGGR	2	5.47	3
							LEEAGGATSAQIELNK	2	5.30	2
							VLIESTELNDDQYR	1	4.68	2
							QLEEAESQVSQLSK	2	4.46	2
							QIEEAEEIAALNLAK	2	4.37	2
							ALDSMQASLEAEAK	1	4.34	2
							GAYEEGQEQLEAVR	2	4.33	2
							LAEAEETIESLNQK	2	4.20	2
							ALDSmQASLEAEAK	2	4.07	2
							LSTEVEDLQLEVDR	2	4.06	2
							LKGAYEEGQEQLEAVR	1	3.95	3
							IEELEEEVEAER	1	3.90	2
							GDIVSVGLQGGEVR	1	3.89	2
							QAEQELADAHEQLNEVSAQNASISAAK	1	3.86	3
							LAGADIETYLLEK	1	3.76	2
							NVQQVTNSIGALcK	2	3.75	2
							NLNDEIAHQDELINK	1	3.69	2
							KADQEISGLKK	1	3.68	3
							VIAYFATVGASK	1	3.61	2
							LTQEAVADLER	2	3.58	2
							KAQQELEEAEER	1	3.55	2
							DIEDLELNVQK	2	3.51	2
							KAEELHAAEVK	1	3.33	3
							ANALQNELEESR	1	3.30	2
							LADELRAEQDHAQTQEK	1	3.27	3
							VDDLAAELDASQK	1	3.26	2
							ISLTTQLEDTKR	2	3.26	2
							ELENELDGEQR	2	3.25	2
							LSIENSDLLR	1	3.23	2
							RANALQNELEESR	2	3.21	2
							DIQTALEEEQR	2	3.20	2
							EEQAEQDGEEEGGR	1	3.18	2
							NKDPLNDTVVDQFK	2	3.16	2
							QLQHTLNEVQSK	2	3.15	2
							LEDEQVVVLK	1	3.14	2
							TALLDSLSGEK	1	2.97	2
							ATSVRPQFDGLAFPPR	1	2.96	3
							TGEELQAAEDK	1	2.94	2
							TGEELQAAEDKINHLNK	1	2.78	3
							IQEKEEEFENTR	1	2.70	3
							SEELEEAKR	1	2.61	2
							TLLEQADR	1	2.53	2
							DKELSSITAK	1	2.50	2
							ELEQTIQR	1	2.47	2
							EQLNSLmTTLR	1	2.43	2
							EGYLLGEIK	1	2.41	2
							LTQEEDAR	2	2.33	2
							LDEAEANALK	1	2.25	2
							VRELENELDGEQR	1	2.22	2
							cNETLDTQQK	2	2.15	2
							TLNDFDASK	1	2.11	2
							NDLENQLR	1	2.09	2
							AGVDHITNEK	1	2.43	3
							DLLDQIGEGGR	1	2.71	2
							SKYESDGVAR	1	2.41	2
							AEELHAAEVK	1	2.37	2
							GALQDYQER	1	1.99	2
							NLEHDLDNLR	1	1.59	2
							GREEQAEQDGEEEGGR	2	5.47	3
							LEEAGGATSAQIELNK	2	5.30	2
							VLIESTELNDDQYR	1	4.68	2
							QLEEAESQVSQLSK	2	4.46	2
							QIEEAEEIAALNLAK	2	4.37	2
							ALDSMQASLEAEAK	1	4.34	2
							GAYEEGQEQLEAVR	2	4.33	2
							LAEAEETIESLNQK	2	4.20	2
							ALDSmQASLEAEAK	2	4.07	2
							LSTEVEDLQLEVDR	2	4.06	2
							LKGAYEEGQEQLEAVR	1	3.95	3
							IEELEEEVEAER	1	3.90	2
							QAEQELADAHEQLNEVSAQNASISAAK	1	3.86	3
							LAGADIETYLLEK	1	3.76	2
							NVQQVTNSIGALcK	2	3.75	2
							NLNDEIAHQDELINK	1	3.69	2
							KADQEISGLKK	1	3.68	3
							VIAYFATVGASK	1	3.61	2
							LTQEAVADLER	2	3.58	2
							KAQQELEEAEER	1	3.55	2
							DIEDLELNVQK	2	3.51	2
							KAEELHAAEVK	1	3.33	3
							ANALQNELEESR	1	3.30	2
							LADELRAEQDHAQTQEK	1	3.27	3

22946664	myosin heavy chain, isoform D (MHC)	245.48	31.96	57	77	224.35	VDDLAAELDASQK	1	3.26	2
							ISLTTQLEDTKR	2	3.26	2
							ELENELDGEQR	2	3.25	2
							LSIENSDLLR	1	3.23	2
							RANALQNELEESR	2	3.21	2
							DIQTALEEEQR	2	3.20	2
							EEQAEQDGEEEGGR	1	3.18	2
							NKDPLNDTVVDQFK	2	3.16	2
							QLQHTLNEVQSK	2	3.15	2
							LEDEQVVVLK	1	3.14	2
							GDIVSVGLQGGETR	1	3.05	2
							TALLDSLSGEK	1	2.97	2
							ATSVRPQFDGLAFPPR	1	2.96	3
							TGEELQAAEDK	1	2.94	2
							TGEELQAAEDKINHLNK	1	2.78	3
							IQEKEEEFENTR	1	2.70	3
							SEELEEAKR	1	2.61	2
							TLLEQADR	1	2.53	2
							DKELSSITAK	1	2.50	2
							ELEQTIQR	1	2.47	2
							EQLNSLmTTLR	1	2.43	2
							EGYLLGEIK	1	2.41	2
							LTQEEDAR	2	2.33	2
							LDEAEANALK	1	2.25	2
							VRELENELDGEQR	1	2.22	2
							cNETLDTQQK	2	2.15	2
							TLNDFDASK	1	2.11	2
							NDLENQLR	1	2.09	2
							AGVDHITNEK	1	2.43	3
							DLLDQIGEGGR	1	2.71	2
							SKYESDGVAR	1	2.41	2
							AEELHAAEVK	1	2.37	2
							GALQDYQER	1	1.99	2
							NLEHDLDNLR	1	1.59	2
22946662	myosin heavy chain, isoform J (MHC)	243.86	31.79	57	77	224.27	GREEQAEQDGEEEGGR	2	5.47	3
							LEEAGGATSAQIELNK	2	5.30	2
							QLEEAESQVSQLSK	2	4.46	2
							QIEEAEEIAALNLAK	2	4.37	2
							ALDSMQASLEAEAK	1	4.34	2
							GAYEEGQEQLEAVR	2	4.33	2
							LAEAEETIESLNQK	2	4.20	2
							ALDSmQASLEAEAK	2	4.07	2
							LSTEVEDLQLEVDR	2	4.06	2
							LKGAYEEGQEQLEAVR	1	3.95	3
							IEELEEEVEAER	1	3.90	2
							GDIVSVGLQGGEVR	1	3.89	2
							QAEQELADAHEQLNEVSAQNASISAAK	1	3.86	3
							LAGADIETYLLEK	1	3.76	2
							NVQQVTNSIGALcK	2	3.75	2
							NLNDEIAHQDELINK	1	3.69	2
							KADQEISGLKK	1	3.68	3
							VIAYFATVGASK	1	3.61	2
							LTQEAVADLER	2	3.58	2
							KAQQELEEAEER	1	3.55	2
							DIEDLELNVQK	2	3.51	2
							KAEELHAAEVK	1	3.33	3
							ANALQNELEESR	1	3.30	2
							LADELRAEQDHAQTQEK	1	3.27	3
							VDDLAAELDASQK	1	3.26	2
							ISLTTQLEDTKR	2	3.26	2
							ELENELDGEQR	2	3.25	2
							LSIENSDLLR	1	3.23	2
							RANALQNELEESR	2	3.21	2
							DIQTALEEEQR	2	3.20	2
							EEQAEQDGEEEGGR	1	3.18	2
							NKDPLNDTVVDQFK	2	3.16	2
							QLQHTLNEVQSK	2	3.15	2
							LEDEQVVVLK	1	3.14	2
							TALLDSLSGEK	1	2.97	2
							ATSVRPQFDGLAFPPR	1	2.96	3
							TGEELQAAEDK	1	2.94	2
							TGEELQAAEDKINHLNK	1	2.78	3
							IQEKEEEFENTR	1	2.70	3
							SEELEEAKR	1	2.61	2
							TLLEQADR	1	2.53	2
							DKELSSITAK	1	2.50	2
							ELEQTIQR	1	2.47	2
							EQLNSLmTTLR	1	2.43	2
							EGYLLGEIK	1	2.41	2
							LTQEEDAR	2	2.33	2
							LDEAEANALK	1	2.25	2
							FIDLPEDQYR	1	2.23	2
							VRELENELDGEQR	1	2.22	2
							cNETLDTQQK	2	2.15	2
							TLNDFDASK	1	2.11	2

							NDLENQLR	1	2.09	2
							AGVDHITNEK	1	2.43	3
							DLLDQIGEGGR	1	2.71	2
							SKYESDGVAR	1	2.41	2
							AEELHAAEVK	1	2.37	2
							GALQDYQER	1	1.99	2
							NLEHDLDNLR	1	1.59	2
61678343	zipper, isoform C (ZIP)	205.23	29.12	50	63	226.78	GTLEAENADLATELR	2	5.02	2
							AQNEELEDDLQLTEDAK	1	4.95	2
							NNDQATLPDNTVAQK	1	4.95	2
							NQFNDPATQAEWTQK	1	4.57	2
							KIETEVADLKEQLNER	1	4.47	3
							LQQEAENITNQLEEAELK	2	4.31	3
							MIQALELDSNLYR	1	4.30	2
							RVQVDEmQAQLAK	1	4.17	3
							TLQNELDDLANTQGTADK	1	4.13	2
							GVADFAIVHYAGR	2	4.11	2
							ANDLSQTLAEEEEKAK	2	4.07	2
							IANLEEQLENEGKER	1	3.84	2
							IQSELEDATIELEAQR	1	3.82	2
							ANDLSQTLAEEEEK	2	3.80	2
							IDAPLVLDQLR	2	3.78	2
							mIQALELDSNLYR	1	3.78	2
							AAETERDELAEEIANNANK	1	3.76	3
							IANLEEQLENEGK	1	3.72	2
							IVSAVLLFGSMK	1	3.68	2
							VQVDEmQAQLAK	1	3.59	2
							AISEQIAQER	1	3.49	2
							REEELTQTLLR	1	3.38	2
							SLEEETVNHEGVLADMR	2	3.35	3
							SLEEETVNHEGVLADmR	1	3.31	3
							EDQSILcTGESGAGK	1	3.26	2
							VQVDEMQAQLAK	1	3.25	2
							LAEIETAQR	1	3.22	2
							AAKEELQALSK	1	3.19	3
							KQAESQIAELQVK	1	3.18	3
							ALESQLAELK	1	3.15	2
							IVSAVLLFGSmK	2	3.10	2
							TGGIGLSSSR	1	3.06	2
							ELIAQNDRLDK	1	3.04	2
							EQVEFAVEAIAK	1	3.00	2
							IAHLLGLSVTDmTR	1	2.89	2
							KQELEDmmQELETR	2	2.84	3
							KYEEDLALTDDQNQK	1	2.80	3
							HSQELNSINDQLENLRK	1	2.79	3
							RLQQLNAIR	1	2.64	2
							YELLTPNVIPK	1	2.56	2
							DLLAKEEGAEEK	1	2.50	2
							EHGDEVEVELAETGKR	1	2.50	3
							EEELTQTLLR	1	2.40	2
							DLETELDEER	1	2.33	2
							cNGVLEGIR	1	1.97	2
							NmLGDREDQSILcTGESGAGK	1	1.94	3
							EHGDEVEVELAETGK	1	2.54	3
							RAAETERDELAEEIANNANK	1	2.97	3
							LVQKEDELK	1	2.38	2
							VIQFLAYVAASKPK	1	2.00	3
							DAEIVGmAQQALTDTQFGAR	1	2.98	3
							ATIATLEAK	1	2.29	2
							IEEEEER	1	2.21	2
							DALRDAEEAK	1	2.08	2
							GTLEAENADLATELR	2	5.02	2
							AQNEELEDDLQLTEDAK	1	4.95	2
							NNDQATLPDNTVAQK	1	4.95	2
							KIETEVADLKEQLNER	1	4.47	3
							LQQEAENITNQLEEAELK	2	4.31	3
							MIQALELDSNLYR	1	4.30	2
							RVQVDEmQAQLAK	1	4.17	3
							TLQNELDDLANTQGTADK	1	4.13	2
							GVADFAIVHYAGR	2	4.11	2
							ANDLSQTLAEEEEKAK	2	4.07	2
							AKHEATITELEER	1	3.95	3
							IANLEEQLENEGKER	1	3.84	2
							IQSELEDATIELEAQR	1	3.82	2
							ANDLSQTLAEEEEK	2	3.80	2
							IDAPLVLDQLR	2	3.78	2
							mIQALELDSNLYR	1	3.78	2
							AAETERDELAEEIANNANK	1	3.76	3
							IANLEEQLENEGK	1	3.72	2
							IVSAVLLFGSMK	1	3.68	2
							VQVDEmQAQLAK	1	3.59	2
							AISEQIAQER	1	3.49	2

1141790	nonmuscle myosin-II heavy chain (ZIP)	204.62	29.01	50	63	226.79	REEELTQTLLR	1	3.38	2
							SLEEETVNHEGVLADMR	2	3.35	3
							SLEEETVNHEGVLADmR	1	3.31	3
							EDQSILcTGESGAGK	1	3.26	2
							VQVDEMQAQLAK	1	3.25	2
							LAEIETAQR	1	3.22	2
							AAKEELQALSK	1	3.19	3
							KQAESQIAELQVK	1	3.18	3
							ALESQLAELK	1	3.15	2
							IVSAVLLFGSmK	2	3.10	2
							TGGIGLSSSR	1	3.06	2
							ELIAQNDRLDK	1	3.04	2
							EQVEFAVEAIAK	1	3.00	2
							IAHLLGLSVTDmTR	1	2.89	2
							KQELEDmmQELETR	2	2.84	3
							KYEEDLALTDDQNQK	1	2.80	3
							HSQELNSINDQLENLRK	1	2.79	3
							RLQQLNAIR	1	2.64	2
							YELLTPNVIPK	1	2.56	2
							DLLAKEEGAEEK	1	2.50	2
							EHGDEVEVELAETGKR	1	2.50	3
							EEELTQTLLR	1	2.40	2
							DLETELDEER	1	2.33	2
							cNGVLEGIR	1	1.97	2
							NmLGDREDQSILcTGESGAGK	1	1.94	3
							EHGDEVEVELAETGK	1	2.54	3
							RAAETERDELAEEIANNANK	1	2.97	3
							LVQKEDELK	1	2.38	2
							VIQFLAYVAASKPK	1	2.00	3
							DAEIVGmAQQALTDTQFGAR	1	2.98	3
							ATIATLEAK	1	2.29	2
							IEEEEER	1	2.21	2
							DALRDAEEAK	1	2.08	2
25009807	AT15526p	15.72	12.21	5	5	48.06	GFAFVEFDDYDAVDK	1	4.13	2
							EYFLQFGNVVSVK	1	4.01	2
							LFIGGLAPYTTEENLK	1	2.93	2
							VVDVVVmR	1	2.39	2
							KLFIGGLAPYTTEENLK	1	2.26	3
158769	ubiquitin	9.72	38.67	2	4	8.43	TITLEVEPSDTIENVK	3	3.84	2
							IQDKEGIPPDQQR	1	1.95	3
160285817	Chain F, Drosophila Nucleosome Core	8.12	27.45	3	3	11.24	VFLENVIR	1	2.78	2
							ISGLIYEETR	1	2.72	2
							DAVTYTEHAK	1	2.61	2
124248356	IP06413p	7.18	3.18	1	3	42.24	EEKTQLEEIKTK	3	2.51	2
259013615	AT04852p	4.86	2.56	1	2	44.26	VLITTDLLAR	2	2.50	2
283046850	MIP14339p	4.45	3.64	1	2	29.60	QFGSPKMTQR	2	2.25	2
158743	beta-2 tubulin	4.36	5.38	2	2	49.81	AILVDLEPGTmDSVR	1	2.52	2
							YLTVAAIFR	1	1.84	2
8067	H2A histone	2.65	14.29	1	1	6.71	AGLQFPVGR	1	2.65	2
112683	14-3-3 protein zeta	2.61	4.03	1	1	28.21	DSTLImQLLR	1	2.61	2
1542877	orf	2.29	0.94	1	1	172.37	DEIVNQQKNVSKSK	1	2.29	2
1150726	transcription factor	2.28	0.77	1	1	112.84	SLVVANTK	1	2.28	2
84795308	CG33309, isoform B	2.26	3.08	1	1	70.76	NQAAKHFHLYLNTKNVEEK	1	2.26	3
158529802	histone H2B	1.97	11.54	1	1	8.84	LLLPGELAK	1	1.97	2
21064361	RE12057p	1.89	2.93	1	1	37.82	GYSFTTTAER	1	1.89	2
296531498	MIP20585p	1.67	2.54	1	1	44.83	QSERELGNIR	1	1.67	2
303228017	RE61743p	-	38.64	1	1	5.20	mcYKEVTYQTNILLYQK	1	1.45	3

PRM										
Accession	Description	Score	Coverage	# Peptides	# PSMs	MW [kDa]	Sequence	# PSMs	XCorr	Charge
10959	paramyosin (PRM)	341.30	51.25	46	111	102.16	INELTTANVSLVSIK	5	5.28	2
							LEEAEGGAEHQFEANR	5	5.17	2
							RQLSEQEGVSQQTTTR	3	4.54	3
							LEEVELNAVAGSK	7	4.47	2
							LAEKDEEIEAIRK	2	4.38	2
							RLAEKDEEIEAIR	1	4.31	2
							ITELEEHIESLIVK	1	4.20	2
							ELEAAEDRADTAESSLNIIR	1	4.14	3
							LDETIILYETSQR	2	4.11	2
							QLQATLDQYAVAQR	1	3.83	2
							HVVEQVHEEQER	4	3.73	2
							QLSEQEGVSQQTTTR	5	3.70	2
							ADLSVQVIQmSER	2	3.60	2
							KQTSIEIEQLNAR	1	3.58	3
							TFVTTSTVPGSQVYIQETTR	2	3.54	2
							LASEVEVLIIDLEK	1	3.49	2
							LLQDDLEVER	9	3.45	2
							QTSIEIEQLNAR	5	3.43	2
							RTVELQYEEAASR	1	3.40	2
							LAEKDEEIEAIR	2	3.29	3
							EVLVQcEEDQK	3	3.24	2
							NLILLQDALDK	3	3.23	2
							LSQENIELTK	3	3.15	2
							TVELQYEEAASR	5	3.14	2
							LSQENIELTKDVQDLK	1	3.13	3
							ADTAESSLNIIR	4	3.09	2
							LGDDLHEAK	2	3.04	2
							LEVSISELNVK	1	3.02	2
							IRDLELELEEEK	3	3.00	2
							IRDLELELEEEKR	1	2.96	3
							ANADATSWQNK	1	2.85	2
							LHELELELR	1	2.76	3
							QSLQLTELQAHYEDVQR	1	2.73	3
							SQVISQLEDAR	1	2.63	2
							SQVISQLEDARR	1	2.51	2
							RLENERDELTAAYK	1	2.51	2
							LEQELSVVASDYEEVSK	1	2.49	3
							LENERDELTAAYK	2	2.49	3
							LAADFNQYR	5	2.27	2
							NQLEEESEAR	4	2.13	2
							LLEDVHLESEETTLLLK	2	2.38	3
							EKADLSVQVIQmSER	1	3.62	2
							KLGDDLHEAK	1	3.18	2
							RLAGLNGELEEVR	1	3.08	2
							WNSEVAAR	1	2.33	2
							VQLDTVSFSK	1	2.18	2
25013082	SD02426p	9.79	4.78	2	4	55.46	LEcYEcSVcSK	2	2.77	2
							QTLPISTEEEETR	2	2.63	2
25009807	AT15526p	5.06	6.45	2	2	48.06	GFAFVEFDDYDAVDK	1	2.50	2
							EYFLQFGNVVSVK	1	2.56	2
157400461	CG30259, isoform A	-	3.52	1	1	63.15	LmSVESYHALEFLQETVAK	1	1.90	3

CG7229/tBRD-2, PSI										
Accession	Description	Score	Coverage	# Peptides	# PSMs	MW [kDa]	Sequence	# PSMs	XCorr	Charge
66771707	IP14655p (CG7229/tBRD-2)	102.98	24.18	16	41	74.59	ALQSQLVNITR	2	4.08	2
							LQSITNQTDAmTR	4	3.64	2
							KLQSITNQTDAMTR	1	3.49	2
							KLQSITNQTDAmTR	3	3.43	2
							SVNEFcLHVDGcFR	2	3.27	2
							NLPINSNPLQmQPIR	1	3.07	2
							SLVETFcDTLNK	2	2.96	2
							QIISNcFLFNR	2	2.87	2
							LQSITNQTDAMTR	1	2.85	2
							TNAPSSAQTER	4	2.73	2
							cGPLINFR	2	2.65	2
							GLATDTMImmK	1	2.53	2
							GLATDTMIMMK	1	2.51	2
							VQPEFIPHPGMAGR	1	2.48	2
							VQPEFIPHPGmAGR	2	2.46	2
							SGDVVYR	1	2.33	2
							HLLDEAR	1	2.24	2
							GLATDTMImMK	3	2.20	2
							GMPSGPEVPcNR	1	1.99	2
							GLATDTmImmK	2	1.96	2
							GmPSGPEVPcNR	2	1.88	2
							NNTANNVVR	1	2.29	2
							VQNNYYK	1	2.49	2
162944872	LD35640p (PSI)	97.72	24.87	18	38	81.54	LAASAGTScEEQIR	1	4.08	2
							LINQQSGAHTEMDR	4	3.53	2
							ISGEAQKIEHAK	1	3.44	2
							SVGKIEEAEAIEK	2	3.35	2
							GTTDQVEAAR	2	3.29	2
							LINQQSGAHTEmDR	1	3.19	2
							INMELNVISR	1	3.18	2
							TIDGLIENVMQR	1	3.14	2
							TIDGLIENVmQR	3	3.13	2
							IQPSQQGGGTAGPSPPSSGGGPGFK	1	3.00	3
							INDDGDSGPESK	1	3.00	2
							QMVLDLIAQK	1	2.92	2
							SKGTTDQVEAAR	4	2.86	2
							SSNDTITHIQAESGVK	1	2.82	3
							LPESVAGAFmGR	1	2.75	2
							EAEMIEQQMK	1	2.61	2
							INmELNVISR	1	2.46	2
							VQVMQDQDR	1	2.29	2
							EAEMIEQQmK	2	2.23	2
							QmVLDLIAQK	1	2.18	2
							VQVmQDQDR	2	2.17	2
							EMIQNMANR	1	1.94	2
							cVIQGTR	1	1.94	2
							INDDGDSGPESKR	1	1.85	2
							EmIQNmANR	1	1.57	2
							EAEmIEQQmK	1	1.71	2
160285817	Chain F, Drosophila Nucleosome Core	64.32	45.10	7	22	11.24	KTVTAMDVVYALKR	1	4.70	3
							KTVTAMDVVYALK	2	4.62	2
							TVTAMDVVYALK	2	3.24	2
							ISGLIYEETR	2	3.21	2
							DAVTYTEHAK	5	3.08	2
							DNIQGITKPAIR	7	2.99	2
							TVTAMDVVYALKR	2	2.87	2
							TVTAmDVVYALK	1	2.69	2
190684756	RE61538p	31.17	24.77	12	12	71.95	SNNHTDQPSGQQQLAENVK	1	4.23	3
							VSAIAGDDEQDQNIPR	1	3.61	2
							DLDALIVISK	1	3.10	2
							TVDGVTNIELEEK	1	3.09	2
							ISGETEESVQR	1	2.93	2
							GYSSDIESVR	1	2.85	2
							IIQEIVDK	1	2.57	2
							QQAGTQQQQPSQVQQQQAAQQQQPKPR	1	2.43	3
							TIDAGVcNYSR	1	2.35	2
							AALNAGDASK	1	2.17	2
							MEIDQQLR	1	1.85	2
							AIQESSMGSTQSFPVTR	1	1.55	2
40216002	GH22839p	30.77	27.21	12	12	64.07	SNNHTDQPSGQQQLAENVK	1	4.23	3
							IAGDDEQDQNIPR	1	3.20	2
							DLDALIVISK	1	3.10	2
							TVDGVTNIELEEK	1	3.09	2
							ISGETEESVQR	1	2.93	2
							GYSSDIESVR	1	2.85	2
							IIQEIVDK	1	2.57	2
							QQAGTQQQQPSQVQQQQAAQQQQPKPR	1	2.43	3
							TIDAGVcNYSR	1	2.35	2
							AALNAGDASK	1	2.17	2
							MEIDQQLR	1	1.85	2
							AIQESSMGSTQSFPVTR	1	1.55	2

160285819	Chain H, Drosophila Nucleosome Core	21.90	36.07	4	12	13.56	HAVSEGTKAVTKYTSSK	2	3.53	3
							QVHPDTGISSK	4	2.82	3
							LLLPGELAK	3	2.22	2
							EIQTAVR	3	1.45	2
115646163	GH26310p	21.10	8.21	5	8	86.82	DQSQLVSTAQLNR	2	4.16	2
							SDASAcTLQER	2	3.19	2
							WVTINAEDPAGPR	1	3.09	2
							AIMcScSSYFR	2	2.02	2
							SALSANNIAGLPNKR	1	1.72	3
121989	Histone H2A.v	19.01	18.44	3	8	14.97	AGLQFPVGR	4	2.97	2
							HLQLAIR	3	1.66	2
							GDEELDSLIK	1	2.86	2
2231301	testis-specific RNP-type RNA binding protein	15.04	5.69	4	7	88.03	IATATPGTGAVTADSK	2	3.43	2
							HAAISAIK	1	2.23	2
							LDTDATVTYGEK	1	2.13	2
							QLATMQEYQR	2	2.12	2
							QLATmQEYQR	1	1.45	2
158429128	Chain E, Drosophila Nucleosome Structure	13.48	20.00	4	6	15.25	EIAQDFKTDLR	2	2.68	2
							RVTIMPK	1	2.26	2
							YRPGTVALR	2	2.26	2
							EIAQDFK	1	1.62	2
40353193	GH09258p	9.04	3.39	2	4	76.50	GSVVLVNAMSAEK	1	3.32	2
							GSVVLVNAmSAEK	1	3.07	2
							LPQLVKPGNR	2	2.65	2
22832294	CG8959	8.45	4.67	3	3	90.83	ELNLPLNLPAGGEK	1	3.21	2
							DLQIGSVINVYGR	1	3.18	2
							NNDAIAEGGR	1	2.06	2
40216175	AT20558p	7.27	4.00	3	3	99.69	TTNLLSLQYNR	1	2.66	2
							ALNTLDDALSK	1	2.65	2
							AQDADALLISVLR	1	1.96	2
16648356	LD32873p	5.77	2.22	2	2	100.36	SNILIATDVASR	1	2.99	2
							SEQQVAEIR	1	2.78	2
21428962	GH25858p	5.32	2.75	2	2	91.31	VELDAPGTVEK	1	2.78	2
							ISFSGNSSNAPK	1	2.54	2
21429856	GH05829p	4.24	4.60	3	3	83.17	NVDTQTIPPPR	1	2.40	2
							LINQFNYcER	1	1.84	2
							QTNEGYLcLFTVK	1	1.12	2
158769	ubiquitin	3.61	21.33	1	1	8.43	TITLEVEPSDTIENVK	1	3.61	2
27819874	LP07910p	3.15	1.83	1	1	75.86	QVLAQIGQIEQR	1	3.15	2
25009807	AT15526p	2.69	3.46	1	1	48.06	GFAFVEFDDYDAVDK	1	2.69	2
92109874	IP13374p	2.64	2.56	1	1	57.59	EEAQDFLEEDTVR	1	2.64	2
60678187	AT28040p	2.62	1.58	1	1	72.62	LVVFVTDGLR	1	2.62	2
115646199	IP03738p	2.32	1.96	1	1	45.03	IYVVGALER	1	2.32	2
14039785	reverse transcriptase-like polymerase	2.02	1.98	1	1	121.91	TFDAILGKDTmKGmGAQIDLK	1	2.02	3
71834275	AT16211p	1.94	1.85	1	1	84.30	LAAAGGSPPSGPPK	1	1.94	2
21064641	RE62270p	1.83	1.37	1	1	97.23	SVATLAITTLLK	1	1.83	2
15291197	GH11385p	-	2.73	1	1	78.23	WSDEKLFQAAKAVNVDIYR	1	1.87	3

PSI										
Accession	Description	Score	Coverage	# Peptides	# PSMs	MW [kDa]	Sequence	# PSMs	XCorr	Charge
162944872	LD35640p (PSI)	167.18	25.13	21	66	81.54	ISGEAQKIEHAK	1	4.17	2
							LINQQSGAHTEMDR	4	4.08	2
							LAASAGTScEEQIR	2	4.02	2
							TIDGLIENVmQR	7	3.80	3
							LINQQSGAHTEmDR	2	3.66	2
							SVGKIEEAEAIEK	2	3.50	2
							INDDGDSGPESK	3	3.42	2
							INMELNVISR	2	3.34	2
							SKGTTDQVEAAR	2	3.16	2
							TIDGLIENVMQR	2	3.12	2
							GTTDQVEAAR	5	3.07	2
							INmELNVISR	2	3.04	2
							SSNDTITHIQAESGVK	3	2.90	3
							EAEMIEQQMK	1	2.76	2
							GREmIQNmANR	1	2.71	3
							LPESVAGAFMGR	1	2.69	2
							RINDDGDSGPESK	1	2.68	2
							QmVLDLIAQK	1	2.60	2
							MIIIQDGPNQELIKPLR	2	2.59	3
							QMVLDLIAQK	1	2.59	2
							EAEMIEQQmK	3	2.43	2
							EAEmIEQQmK	1	2.40	2
							IEEAEAIEK	2	2.28	2
							GREMIQNMANR	1	2.24	2
							LPESVAGAFmGR	2	2.18	2
							VQVmQDQDR	5	2.15	2
							EMIQNMANR	2	2.11	2
							cVIQGTR	1	2.02	2
							VQVMQDQDR	2	2.01	2
							EmIQNMANR	1	1.96	2
							IQTEcGcK	1	1.59	2
66771707	IP14655p	37.51	13.35	9	18	74.59	ALQSQLVNITR	2	3.38	2
							LQSITNQTDAMTR	1	3.23	2
							LQSITNQTDAmTR	1	3.15	2
							SLVETFcDTLNK	2	3.12	2
							QIISNcFLFNR	1	2.64	2
							KLQSITNQTDAmTR	2	2.60	2
							TNAPSSAQTER	2	2.50	2
							cGPLINFR	1	2.36	2
							GLATDTMImmK	3	1.71	2
							GLATDTmImmK	2	1.67	2
							GmPSGPEVPcNR	1	1.47	2
20976828	GH10652p	21.01	8.68	6	8	88.16	TVGETETFLTSNEITIK	2	4.24	2
							DLVGVAQTGSGK	1	3.30	2
							LLTDISAENETK	1	3.27	2
							cTYLVLDEADR	2	2.43	2
							GVEIVIATPGR	1	2.09	2
							GLDVDDVK	1	1.42	2
40216002	GH22839p	10.87	5.89	3	4	64.07	IAGDDEQDQNIPR	1	3.53	2
							ISGETEESVQR	2	3.01	2
							GYSSDIESVR	1	1.72	2
158769	ubiquitin	6.40	21.33	1	2	8.43	TITLEVEPSDTIENVK	2	3.26	2
71834275	AT16211p	5.67	3.30	2	2	84.30	LAAAGGSPPSGPPK	1	3.98	2
							QLVAATIScLR	1	1.69	2
16198217	LD41427p	3.31	2.54	1	1	60.58	SLGGFVVNAELEAR	1	3.31	2
21428962	GH25858p	2.17	1.32	1	1	91.31	VELDAPGTVEK	1	2.17	2
84795308	CG33309, isoform B	-	3.08	1	1	70.76	NQAAKHFHLYLNTKNVEEK	1	1.90	3

PABP, HSP68										
Accession	Description	Score	Coverage	# Peptides	# PSMs	MW [kDa]	Sequence	# PSMs	XCorr	Charge
76803808	Polyadenylate-binding protein (PABP)	154.14	45.27	25	53	69.88	GAQPQVQGTHAAAAAANNMR	3	4.91	2
							AKVEEAVAVLQVHR	3	4.89	2
							LIASLLANAKPQEQK	1	4.89	2
							AITGQQTAAPNMQIPGAQIAGGAQQR	2	4.76	3
							SLGYAYVNFQQPADAER	1	4.71	2
							GAQPQVQGTHAAAAAANNmR	2	4.32	3
							AITGQQTAAPNmQIPGAQIAGGAQQR	2	4.22	3
							VEEAVAVLQVHR	2	4.12	2
							GYGFVHFETEEAANTSIDK	2	4.08	3
							VVGSKPLYVALAQR	3	3.67	2
							NPPVPQLHQTQPIPQQLQGK	4	3.51	3
							HESVFGVNLYVK	2	3.36	3
							AIYDTFSAFGNILScK	1	3.32	2
							KAHLASQYMR	2	3.24	3
							RHESVFGVNLYVK	1	3.22	3
							SGVGNVFIK	1	3.19	2
							RSLGYAYVNFQQPADAER	2	2.99	3
							KAHLASQYmR	2	2.84	3
							FSSAGPVLSIR	1	2.74	2
							NFTEDFDDEKLK	1	2.57	2
							LFTNVYVK	1	2.40	2
							NLDDTIDDDRLR	3	2.40	2
							FFGSQVATQMR	1	2.35	2
							NLDDTIDDDR	1	2.33	2
							IAFSPYGNITSAK	1	2.30	2
							ALDTMNFDLVR	1	2.21	2
							FFGSQVATQmR	4	1.81	2
							ALDTmNFDLVR	1	2.26	2
							VNGmLLNGK	1	2.26	2
							KFEELK	1	1.69	2
13124306	Heat shock protein 68 (HSP68)	98.23	38.74	22	35	69.70	IINEPTAAALAYGLDK	2	4.65	2
							MKETAEAYLGTTVK	2	4.23	2
							STAGDTHLGGEDFDNR	2	4.19	2
							GGGDGQQAPNFGQQAGGYK	2	4.04	2
							VQNLLQNFFGGK	1	3.75	2
							mKETAEAYLGTTVK	1	3.46	2
							DAGAIAGINVLR	2	3.43	2
							DNNVLGTFDLTGVPPAPR	2	3.08	2
							VEIIANDQGNR	1	3.00	2
							mLSEAEKYAEEDER	2	3.00	3
							NQLETYLFGVK	1	2.92	2
							ELEQFcSPImTK	2	2.92	2
							LVNHFAEEFKR	1	2.79	2
							ARFEELcGDLFR	1	2.76	2
							TTPSYVAFTDSER	2	2.76	2
							FEELcGDLFR	2	2.58	2
							SQIHDIVLVGGSTR	1	2.39	3
							DAVITVPAYFNDSQR	1	2.34	2
							MLSEAEKYAEEDERHR	1	2.30	3
							LSQADIDR	2	2.16	2
							MLSEAEKYAEEDER	1	2.49	3
							ETAEAYLGTTVK	1	2.02	2
							NTLEPVEK	1	1.94	2
							NQVAMNPK	1	1.82	2
6960212	cytoplasmic protein 89BC	23.14	11.37	5	9	63.05	AKPTDALQGVVLNPK	1	4.00	2
							IGKPTLVGETSR	2	2.89	2
							VKEYEAHIEQAK	1	2.34	3
							AQYQDQLSR	2	2.29	2
							SITGTAAPPLTLTAK	3	2.24	2
25009807	AT15526p	22.86	21.66	8	9	48.06	EYFLQFGNVVSVK	1	3.62	2
							LFIGGLAPYTTEENLK	1	3.09	2
							VVDVVVmR	1	2.87	2
							mNPYSAGPPNSYR	1	2.64	2
							MNPYSAGPPNSYR	1	1.92	2
							DNHDEEcLR	1	1.96	3
							LFYGQWGK	1	1.85	2
							AQENRPHIIDGK	1	2.71	2
45447000	stromal interaction molecule, isoform B	19.73	13.66	5	7	51.89	ITNSTEDLDDESIQGK	2	4.51	2
							NALGDVTNELQER	1	3.23	2
							MEQENVATEK	2	2.03	2
							AEQSLQEMQK	1	2.33	2
							SSLVGAFVSTHGK	1	3.29	2
224983344	Chain A, Sumo-3 (Dsmt3)	17.40	45.45	4	7	9.95	VLGQDNAVVQFK	2	3.73	2
							KGGETEHINLK	2	2.70	3
							AGLSMQVVR	1	2.67	2
							AGLSmQVVR	1	2.63	2
							LmNAYcDR	1	1.41	2
7301065	CG13597	11.60	7.80	3	4	59.21	ATESLDQASTSFSAPR	1	4.59	2
							TESLFVQEQR	2	2.55	2
							YSQNPDYDHDREEK	1	2.07	3

15010456	GH05807p	11.23	4.38	3	4	67.84	LGLQVcAVK	1	2.85	2
							VVDALNATR	2	2.78	2
							GVDIVcNALR	1	2.93	2
19527869	AT11329p	11.06	8.86	3	4	48.94	TVVTTSVPTTFPR	2	3.58	2
							TIAVGcHHQAGSAAPR	1	2.58	3
							IYQPSGSSQR	1	1.99	2
34222759	Protein painting of fourth	7.88	6.06	2	3	55.07	ELTNKPGQTPASEVLLVR	1	3.29	3
							AALESGDGPDAK	2	2.40	2
18446959	AT14039p	4.96	3.85	2	2	65.47	GGVVTLTTDESYR	1	2.88	2
							EIVYVINLR	1	2.08	2
15292111	LD38872p	4.02	3.03	2	2	68.57	QmVLDLIAQK	1	2.02	2
							INmELNVISR	1	2.00	2
66771707	IP14655p	3.07	1.63	1	1	74.59	ALQSQLVNITR	1	3.07	2
16197857	GH12014p	3.02	2.94	1	1	53.38	NGLmAEAGTGGFLR	1	3.02	2
12644007	Maternal protein exuperantia	3.01	2.63	1	1	57.94	NIEGLDIALQSIGR	1	3.01	2
7303284	CG6209	2.73	1.81	1	1	68.98	FAEQIAIEEAK	1	2.73	2
17862172	LD11291p	2.50	1.77	1	1	76.07	EYIASSEQENLK	1	2.50	2
23096163	CG32100	2.19	3.08	1	1	54.47	EPSSDSTPTPGQAQR	1	2.19	2
13124721	Succinate dehydrogenase [ubiquinone] flavoprotein subunit, mitochondrial	2.16	1.66	1	1	72.30	DGPILQDGVNK	1	2.16	2
6466460	Hsp90-related protein TRAP1	2.11	1.75	1	1	77.37	ELISNASDALEK	1	2.11	2
15291295	GH14707p	2.10	1.44	1	1	72.73	NFLVVGDWR	1	2.10	2
33636647	LD04013p	2.07	2.05	1	1	56.74	TGDPIDSVLER	1	2.07	2
27819855	RE03224p	2.06	2.06	1	1	59.57	SPEVNTVQTEK	1	2.06	2
60678095	LD10516p	-	2.46	1	1	89.50	GMSEQEIESILDKTmVLFR	1	1.93	3
60677991	LP16040p	-	2.49	1	1	63.76	LQHLEQQNEEYSSR	1	1.83	3
51092077	RE33235p	-	2.49	1	1	92.29	NMVFSHIEYQLQLPKYYmLRK	1	0.66	3
17861788	GH27293p	-	2.63	1	1	63.80	YPSAESKQESEEDER	1	1.59	2
7501	unnamed protein product	-	11.90	1	1	9.02	AGFAGDDAPR	1	1.52	2

tBRD-1

Accession	Description	Score	Coverage	# Peptides	# PSMs	MW [kDa]	Sequence	# PSMs	XCorr	Charge
7301065	CG13597 (tBRD-1)	147.74	34.89	21	50	59.21	KATESLDQASTSFSAPR	4	5.61	2
							ATESLDQASTSFSAPR	6	4.77	2
							mESIDLSTEVELKPK	2	4.39	2
							LDSDNFESFDGFVSSVR	2	4.24	2
							YYWQASEALEDFK	1	3.93	2
							YSQNPDYDHDREEK	3	3.69	2
							RYSQNPDYDHDREEK	1	3.54	3
							LLMEAFYMR	1	3.16	2
							HPVDSVSLGVPDYHAVVK	3	3.04	3
							LLMEAFYmR	1	2.97	2
							QVTWAFNQADYWR	2	2.84	2
							KTNEIFEKR	1	2.83	3
							TESLFVQEQR	8	2.82	2
							LLmEAFYmR	2	2.77	2
							YSQNPDYDHDR	2	2.44	2
							MESIDLSTEVELKPK	1	2.38	3
							TNEIFEK	1	2.32	2
							KMFQNALR	1	2.22	2
							HPmDLSTIR	1	2.05	2
							TNILEELK	1	1.96	2
							HPMDLSTIR	1	1.82	2
							SHSDHLLK	1	1.65	2
							mFQNALR	1	2.38	2
							YRELIATAK	1	2.30	2
							RTNILEELK	1	2.25	2
							KmFQNALR	1	2.27	2
6960212	cytoplasmic protein 89BC	34.79	17.15	8	13	63.05	AKPTDALQGVVLNPK	1	4.13	2
							IGKPTLVGETSR	2	3.31	2
							NVLMHGPPGTGK	1	3.27	2
							SITGTAAPPLTLTAK	3	3.20	2
							VKEYEAHIEQAK	2	3.02	3
							YEDQLLQQQR	2	2.60	2
							AQYQDQLSR	1	2.58	2
							FKLDTFDYGK	1	1.49	2
25009824	AT17665p	16.75	13.65	6	7	55.32	SVFTNNNPSPQATmR	2	3.38	2
							VGIFNAEDFLSR	1	2.68	2
							ITQAADGLVK	1	2.39	2
							TTTDTFTDLTK	1	2.35	2
							FVSSmNSPLAPR	1	2.20	2
							VEIVTYDGMK	1	1.64	2
21392176	RE66761p	15.75	13.75	7	7	66.35	AALYIQLDQR	1	3.16	2
							NAAVAQINVTK	1	3.07	2
							QAISLDGTAPDQELER	1	3.01	2
							EDcTASLEFNPR	1	2.51	2
							GIADFAEAER	1	1.99	2
							TDMAIFYQNR	1	2.00	2
							SQAELVHVYSLR	1	1.39	3
34222759	Protein painting of fourth	11.55	10.91	5	5	55.07	AGADVEATIcDINR	1	2.94	2
							DVVLPISVAPR	1	2.40	2
							AALESGDGPDAK	1	2.30	2
							ELVDSPLNSR	1	2.29	2
							FGTGHLR	1	1.62	2
20151275	AT09290p	10.07	8.31	3	4	45.90	NQAAITIQAAWR	1	3.85	2
							GADGTELLYQYFR	2	3.16	2
							FVDAYmSAER	1	1.54	2
12644007	Maternal protein exuperantia	9.06	10.34	4	4	57.94	NIEGLDIALQSIGR	1	3.40	2
							DARPSSSPSASTEFGAGGDK	1	3.11	3
							SVSSLPDSTTK	1	2.56	2
							ELFDGNASVR	1	1.58	2
283046850	MIP14339p	8.17	3.64	1	4	29.60	QFGSPKMTQR	3	2.09	2
							QFGSPKmTQR	1	2.17	2
10728769	CG7311, isoform A	6.16	4.46	2	2	73.83	TALIEAEDFASGSSSR	1	3.97	2
							AVINATGSSTDAIR	1	2.19	2
22759458	ATP synthase-beta, isoform B	5.51	9.45	1	2	13.37	TIAmDGTEGLVR	2	2.80	2
5921205	ATP synthase subunit alpha, mitochondrial	5.09	2.54	1	2	59.38	GIRPAINVGLSVSR	2	2.64	3
1813955	succinate dehydrogenase flavoprotein subunit	4.68	2.55	1	2	54.91	ENAGEASVANLDK	2	2.19	2
23170725	CG8036, isoform D	4.53	2.24	1	2	63.00	TGAAPDVDINNIK	2	2.30	2
158769	ubiquitin	3.71	21.33	1	1	8.43	TITLEVEPSDTIENVK	1	3.71	2
220903213	CG11913	3.40	2.29	1	1	60.11	LDEVEDVVTDNR	1	3.40	2
269996039	MIP14116p	2.67	1.26	1	1	114.29	SRNSNSNTLNRGR	1	2.67	2
187403726	multiplexin transcript 8	2.66	1.20	1	1	86.50	SPYRVLLPEK	1	2.66	3
15292137	LD39959p	2.63	1.81	1	1	50.21	IFNTYGPR	1	2.63	2
74868947	Eukaryotic translation initiation factor 3 subunit D-2	2.53	3.99	1	1	62.71	GTPNIASLGGSTAGGATASTTK	1	2.53	2
9887208	Stretchin-MLCK	2.46	8.61	1	1	23.54	mHNIFFSIDILNKSRMDK	1	2.46	3
84795308	CG33309, isoform B	2.45	3.08	1	1	70.76	NQAAKHFHLYLNTKNVEEK	1	2.45	3
259013615	AT04852p	2.26	2.56	1	1	44.26	VLITTDLLAR	1	2.26	2
5911298	BcDNA.LD28120	2.10	1.83	1	1	72.88	LSLTSESAGGAR	1	2.10	2
552090	epidermal growth factor receptor type I	2.07	8.62	1	1	20.08	IcIGTKSRLSVPSNK	1	2.07	2
20151329	GH03947p	2.02	0.69	1	1	137.43	ATPDRLNSR	1	2.02	2
25009807	AT15526p	1.81	3.69	1	1	48.06	LFIGGLAPYTTEENLK	1	1.81	2
7501	unnamed protein product	1.75	11.90	1	1	9.02	AGFAGDDAPR	1	1.75	2
969079	ORF1; similar to human alpha-actinin 3	1.69	2.33	1	1	58.96	SIGVSDSVDLIR	1	1.69	2

220902047	CG42231	1.67	3.31	1	1	27.63	NISAEQLR	1	1.67	2
102230389	DSC1	-	0.75	1	1	273.83	LAKSGSGGSNTPmAPTPK	1	1.75	3

Rb97D, beta 1-Tubulin, beta 2-Tubulin

Accession	Description	Score	Coverage	# Peptides	# PSMs	MW [kDa]	Sequence	# PSMs	XCorr	Charge
25009807	AT15526p (Rb97D)	482.52	40.55	24	208	48.06	GFAFVEFDDYDAVDK	10	4.54	2
							EYFLQFGNVVSVK	15	4.50	2
							LFVGGLKDNHDEEcLR	2	4.46	2
							RGFAFVEFDDYDAVDK	3	4.00	2
							LFIGGLAPYTTEENLK	14	3.81	2
							AQENRPHIIDGKTVEAK	1	3.44	3
							mNPYSAGPPNSYR	22	3.33	2
							AQENRPHIIDGK	11	3.18	2
							DEPLSDESADVIVLADR	1	3.12	2
							VVDVVVmR	18	3.09	2
							VVDVVVMR	30	3.03	2
							DNHDEEcLR	6	2.93	3
							KLFIGGLAPYTTEENLK	5	2.93	3
							MNPYSAGPPNSYR	16	2.87	2
							EEDDIcELEHLR	1	2.81	2
							KSIYNLDKK	2	2.78	2
							LFYGQWGK	18	2.46	2
							YVHVDVK	3	2.37	2
							ETNISVKK	2	2.29	2
							YVHVDVKK	2	2.24	2
							SIYNLDKK	7	2.22	2
							GFGFITYTK	5	2.22	2
							LFVGGLK	4	1.73	2
							KLFVGGLKDNHDEEcLR	1	2.29	3
							SLmVDR	3	1.47	2
							SLMVDR	4	1.45	2
							ETNISVK	2	1.11	2
158743	beta-2 tubulin	189.23	48.88	20	64	49.81	mSmKEVDEQMLNIQNK	4	4.68	3
							mSmKEVDEQmLNIQNK	1	4.53	3
							MSATFIGNSTAIQELFK	2	4.48	2
							EIVHIQAGQcGNQIGGK	4	4.42	2
							IMNTFSVVPSPK	5	4.16	2
							mSATFIGNSTAIQELFK	2	3.93	2
							EVDEQMLNIQNK	3	3.75	2
							mREIVHIQAGQcGNQIGGK	1	3.74	3
							EVDEQmLNIQNK	3	3.61	2
							ImNTFSVVPSPK	3	3.61	2
							LAVNMVPFPR	1	3.60	2
							LAVNmVPFPR	1	3.56	2
							INVYYNEATGAK	1	3.47	2
							AILVDLEPGTMDSVR	1	3.43	2
							NSSFFVEWIPNNcK	1	3.37	2
							VSEQFTAMFR	1	3.32	2
							LHFFmPGFAPLTSR	5	3.26	2
							VSEQFTAmFR	1	3.18	2
							AILVDLEPGTmDSVR	3	3.14	2
							KLAVNmVPFPR	2	3.05	2
							FPGQLNADLRK	2	2.69	2
							TAVcDIPPR	4	2.50	2
							ALTVPELTQQMFDAK	2	2.49	2
							ALTVPELTQQmFDAK	1	2.40	2
							LHFFMPGFAPLTSR	1	2.39	2
							NMMAAcDPR	1	2.30	2
							YLTVAAIFR	4	2.16	2
							GHYTEGAELVDSVLDVVR	1	2.93	3
							NMmAAcDPR	2	2.28	2
							IREEYPDR	1	2.48	2
158739	beta-1 tubulin	124.64	34.68	14	44	50.12	mSmKEVDEQMLNIQNK	4	4.68	3
							mSmKEVDEQmLNIQNK	1	4.53	3
							MSATFIGNSTAIQELFK	2	4.48	2
							mSATFIGNSTAIQELFK	2	3.93	2
							EVDEQMLNIQNK	3	3.75	2
							EVDEQmLNIQNK	3	3.61	2
							LAVNMVPFPR	1	3.60	2
							LAVNmVPFPR	1	3.56	2
							LHFFmPGFAPLTSR	5	3.26	2
							mREIVHIQAGQcGNQIGAK	1	3.19	3
							KLAVNmVPFPR	2	3.05	2
							FPGQLNADLRK	2	2.69	2
							TAVcDIPPR	4	2.50	2
							ALTVPELTQQMFDAK	2	2.49	2
							ALTVPELTQQmFDAK	1	2.40	2
							LHFFMPGFAPLTSR	1	2.39	2
							NMMAAcDPR	1	2.30	2
							YLTVAAIFR	4	2.16	2
							GHYTEGAELVDSVLDVVR	1	2.93	3
							NMmAAcDPR	2	2.28	2
							IREEYPDR	1	2.48	2

5921205	ATP synthase subunit alpha, mitochondrial	44.27	23.01	11	16	59.38	TGAIVDVPVGDELLGR	1	4.28	2
							VVDALGNAIDGK	1	3.80	2
							SAEISNILEER	1	3.35	2
							HALIIYDDLSK	2	3.18	2
							DGAISEASDAK	3	3.06	2
							GIRPAINVGLSVSR	2	3.03	2
							TSEQALLDTIAK	2	3.00	2
							EAYPGDVFYLHSR	1	2.93	2
							AVDSLVPIGR	1	2.59	2
							VLSIGDGIAR	1	2.41	2
							ELIIGDR	1	1.13	2
17861912	GM14349p	34.22	26.62	10	13	51.82	FYSGFGGQVDFIR	1	3.34	2
							IVGSFLIGDK	1	2.99	2
							SGDTVFAGGAAATPVALLNAmAK	1	2.93	3
							mYELIQIADPK	1	2.91	2
							EPPIVKPEEAVAcVK	1	2.69	2
							SNSFFMGANVR	1	2.67	2
							LIVAQINPK	2	2.60	2
							MYELIQIADPK	2	2.53	2
							TFGDAIIHK	1	2.14	2
							LIAENLVK	1	2.31	2
							AHVHYVVTEHGIASLFGK	1	2.98	3
158030193	skpA associated protein, isoform B	26.51	18.13	8	9	54.77	VVYDPQTAEELSSK	2	3.76	2
							DLNLNmPVVVR	1	3.51	2
							AADLAVHLAQIVK	1	3.30	2
							cDVIAEGIISATK	1	3.21	2
							MDVNFEIPDAQK	1	3.01	2
							MIDQLLVTK	1	2.60	2
							AFNGPVLIASK	1	2.46	2
							FDDNAEFR	1	1.63	2
63108427	AT20029p	25.85	16.51	9	10	57.40	LVVDIATLNTGVK	1	4.54	2
							AGVVVLSDPLLYGQK	1	4.35	2
							TMDWIEAQR	1	3.11	2
							LNAFLMIAK	1	3.08	2
							LVVDIATLNTGVKK	1	2.80	2
							GVVVGLYQK	1	2.60	2
							AEAQNLAR	1	1.87	2
							VAAGVGAR	2	1.65	2
							GITFNSGALNLRPcR	1	1.38	3
287945	ATP synthase beta subunit	20.78	10.40	4	6	53.49	LVLEVAQHLGENTVR	3	4.80	2
							FTQAGSEVSALLGR	1	3.27	2
							IGLFGGAGVGK	1	3.16	2
							TIAmDGTEGLVR	1	2.64	2
15292289	LD45641p	18.61	22.55	6	7	36.48	DAGVLGLSSAAmER	1	3.84	2
							LETEKYDLEER	1	3.83	2
							DAGVLGLSSAAMER	1	3.26	2
							LFEGGWDEISK	1	2.88	2
							AQELWELIVK	1	2.76	2
							IKPLAIEGFGEAK	1	2.05	2
							GLDPEALTGK	1	1.59	2
135410	Tubulin alpha-2 chain	14.36	14.25	5	5	49.93	AVFVDLEPTVVDEVR	1	4.47	2
							DVNAAIATIK	1	3.03	2
							VGINYQPPTVVPGGDLAK	1	2.56	2
							EDAANNYAR	1	1.71	2
							QLFHPEQLITGK	1	2.60	2
16198089	LD30622p	12.84	14.00	4	5	40.45	NGTDAEAISPDAVR	1	3.66	2
							ScLPPTVVDQIK	2	2.72	2
							QGFQFLLLDTR	1	2.46	2
							DIAFLDTYAMSR	1	1.74	2
16768502	GM06507p	12.77	9.77	3	4	48.64	GKENVQLSAQNILScTR	2	4.11	3
							DFFAYSGGVYR	1	2.59	2
							DSLYTVGPAYSLNR	1	2.48	2
25012530	RE33426p	11.34	5.04	2	4	54.74	YPASTVQILGAEK	2	3.32	2
							NLTQDQLEDLEK	2	2.86	2
124248356	IP06413p	10.38	3.18	1	4	42.24	EEKTQLEEIKTK	4	2.79	2
16769538	LD37574p	8.50	15.34	3	3	38.16	GNEAANVTGPSGEPVR	1	3.68	2
							NGAQELQNTTTESTA	1	2.79	2
							GLGGGDGSAEPGVHDQNPEGLQR	1	2.03	3
7292468	CG1309	8.10	6.87	3	3	58.37	RTEDEIIAEDNHIDTNR	1	3.91	3
							NLmAVFLLR	1	2.14	2
							mPAALAGIVK	1	2.06	2
75026227	RuvB-like helicase 2	4.17	4.37	2	2	53.51	FVQcPEGELQK	1	2.51	2
							VYSLFLDENR	1	1.66	2
158769	ubiquitin	3.45	21.33	1	1	8.43	TITLEVEPSDTIENVK	1	3.45	2
16768026	GH12543p	3.18	2.85	1	1	56.69	TTANAVTLDDALGGK	1	3.18	2
8314	otefin	3.08	3.45	1	1	44.63	ASIGGQASPAASPK	1	3.08	2
7243680	pontin	3.05	2.41	1	1	50.18	LSEIGTSSTLR	1	3.05	2
19527953	AT17695p	2.71	4.07	1	1	31.47	FSPNETQALYR	1	2.71	2
300639630	unnamed protein product	2.48	2.51	1	1	46.22	ILcDNITGLSK	1	2.48	2
417037	Protein germ cell-less	2.36	2.11	1	1	65.11	HEHERVLSLQTK	1	2.36	2
295293319	MIP22223p	2.33	2.78	1	1	47.91	VAVGGNYDASER	1	2.33	2
16198163	LD36412p	2.12	2.02	1	1	44.83	ALDFLDNR	1	2.12	2
19527993	AT19426p	2.12	2.62	1	1	38.80	HGIGVYVFK	1	2.12	2
6018892	EG:BACR42I17.5	1.98	3.49	1	1	39.85	SLAGNAAVLIKAR	1	1.98	2
21430612	RE27864p	1.89	1.35	1	1	74.42	TLIDQESVR	1	1.89	2

17861608	GH14951p	1.87	2.48	1	1	64.41	QAFHEmLISELAAMR	1	1.87	2
220902047	CG42231	1.77	3.31	1	1	27.63	NISAEQLR	1	1.77	2
19527877	AT12476p	1.70	3.46	1	1	29.37	mSVEAGAVK	1	1.70	2
290965494	MIP17622p	1.67	1.18	1	2	56.12	QDLINR	2	1.52	2
84795308	CG33309, isoform B	-	3.08	1	1	70.76	NQAAKHFHLYLNTKNVEEK	1	1.16	3
