# Supplementary figures and images for: Two bromodomain proteins functionally interact to recapitulate an essential BRDT-like function in Drosophila spermatocytes
Source: Open Biol. 2015 Feb 4;5(2):140145. doi: 10.1098/rsob.140145 (PMC4345279; doi:10.1098/rsob.140145)

# Kimura et al.\_Supplemental Fig.1

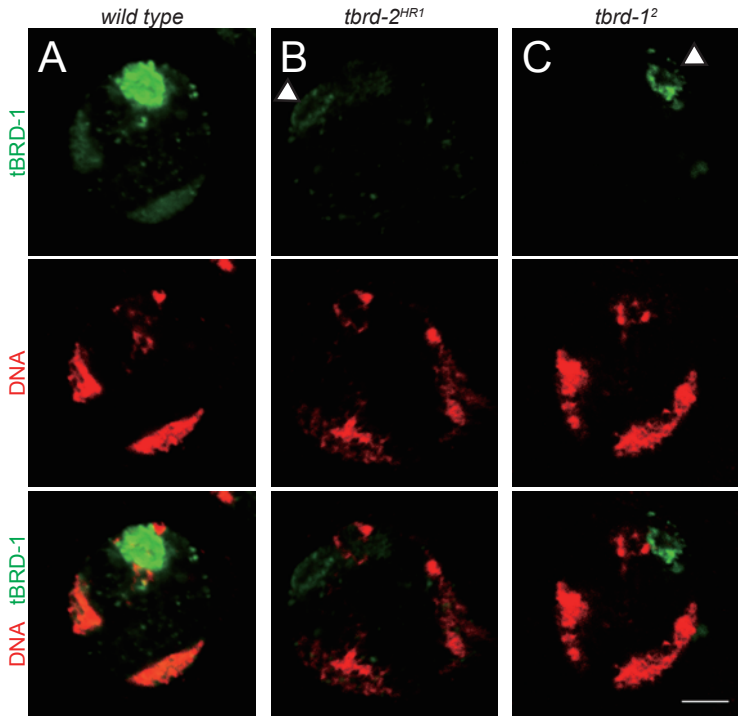

Supplement: Figure S1 [file rsob140145supp3.pdf]
